# *Mycobacterium tuberculosis* whole genome sequencing and protein structure modelling provides insights into anti-tuberculosis drug resistance

**DOI:** 10.1186/s12916-016-0575-9

**Published:** 2016-03-23

**Authors:** Jody Phelan, Francesc Coll, Ruth McNerney, David B. Ascher, Douglas E. V. Pires, Nick Furnham, Nele Coeck, Grant A. Hill-Cawthorne, Mridul B. Nair, Kim Mallard, Andrew Ramsay, Susana Campino, Martin L. Hibberd, Arnab Pain, Leen Rigouts, Taane G. Clark

**Affiliations:** Faculty of Infectious and Tropical Diseases, London School of Hygiene & Tropical Medicine, Keppel Street, London, WC1E 7HT UK; University of Cape Town Lung Institute, Lung Infection & Immunity Unit, Old Main Building, Groote Schuur Hospital, Observatory, Cape Town, 7925 South Africa; Department of Biochemistry, University of Cambridge, 80 Tennis Court Road, Cambridge, CB2 1GA UK; Centro de Pesquisas René Rachou, Fundação Oswaldo Cruz, Avenida Augusto de Lima 1715, Belo Horizonte, 30190-002 Brazil; Mycobacteriology Unit, Institute of Tropical Medicine, Antwerp, Belgium; Pathogen Genomics Laboratory, BESE Division, King Abdullah University of Science and Technology (KAUST), Thuwal, Saudi Arabia; Sydney Emerging Infections and Biosecurity Institute and School of Public Health, Sydney Medical School, University of Sydney, Sydney, NSW 2006 Australia; Special Programme for Research and Training in Tropical Diseases (TDR), World Health Organisation, Geneva, Switzerland; Department of Biomedical Sciences, Antwerp University, Antwerp, Belgium; Faculty of Epidemiology and Population Health, London School of Hygiene & Tropical Medicine, Keppel Street, London, WC1E 7HT UK; Department of Pathogen Molecular Biology, Faculty of Infectious and Tropical Diseases, London School of Hygiene & Tropical Medicine, Keppel Street, London, UK

**Keywords:** Tuberculosis, Drug resistance, Genomics, Protein structural modelling, Association study, Convergent evolution

## Abstract

**Background:**

Combating the spread of drug resistant tuberculosis is a global health priority. Whole genome association studies are being applied to identify genetic determinants of resistance to anti-tuberculosis drugs. Protein structure and interaction modelling are used to understand the functional effects of putative mutations and provide insight into the molecular mechanisms leading to resistance.

**Methods:**

To investigate the potential utility of these approaches, we analysed the genomes of 144 *Mycobacterium tuberculosis* clinical isolates from The Special Programme for Research and Training in Tropical Diseases (TDR) collection sourced from 20 countries in four continents. A genome-wide approach was applied to 127 isolates to identify polymorphisms associated with minimum inhibitory concentrations for first-line anti-tuberculosis drugs. In addition, the effect of identified candidate mutations on protein stability and interactions was assessed quantitatively with well-established computational methods.

**Results:**

The analysis revealed that mutations in the genes *rpoB* (rifampicin), *katG* (isoniazid), *inhA*-promoter (isoniazid), *rpsL* (streptomycin) and *embB* (ethambutol) were responsible for the majority of resistance observed. A subset of the mutations identified in *rpoB* and *katG* were predicted to affect protein stability. Further, a strong direct correlation was observed between the minimum inhibitory concentration values and the distance of the mutated residues in the three-dimensional structures of *rpoB* and *katG* to their respective drugs binding sites.

**Conclusions:**

Using the TDR resource, we demonstrate the usefulness of whole genome association and convergent evolution approaches to detect known and potentially novel mutations associated with drug resistance. Further, protein structural modelling could provide a means of predicting the impact of polymorphisms on drug efficacy in the absence of phenotypic data. These approaches could ultimately lead to novel resistance mutations to improve the design of tuberculosis control measures, such as diagnostics, and inform patient management.

**Electronic supplementary material:**

The online version of this article (doi:10.1186/s12916-016-0575-9) contains supplementary material, which is available to authorized users.

## Background

Tuberculosis, caused by *Mycobacterium tuberculosis* (Mtb), is an important global public health issue (>8.7 million new cases, 1.4 million deaths each year [1]). The *M. tuberculosis* phylogeny *c*onsists of four major lineages (L1 - Indo-Oceanic, L2 - East-Asian, L3 - East-African-Indian, L4 - Euro-American), which may vary in their propensity to transmit and cause disease [2]. The Mtb genome (size 4.4 Mb, GC content 65.5 %) is relatively clonal compared to most other bacteria, with no horizontal transfer, and low mutation and recombination rates [3]. Mtb drug resistance is a serious challenge to effective control [1]. Standard first-line anti-TB therapy involves four drugs (rifampicin [RMP], isoniazid [isonicotinic acid hydrazide] [INH], ethambutol [EM]), pyrazinamide [PZA]), with streptomycin (SM) more commonly used when treatment fails. Resistance to at least RMP and INH is denoted as multi drug-resistance (MDR-TB). It has been estimated that ~4 % of new cases are MDR-TB [1], and without effective treatment can remain a source of transmission [4]. Additional resistance to any fluoroquinolone and second-line injectable drug (e.g. amikacin, kanamycin, capreomycin), is denoted as extensively drug resistance (XDR-TB), and such cases have been reported in 100 countries [1].

In routine diagnostic practice susceptibility to anti-tuberculosis drugs is assessed phenotypically by determining the proportion of bacteria that will grow at critical concentrations of the drug [5]. For most anti-tuberculosis drugs, a single concentration is used, but for some drugs two concentrations are used to indicate high and low levels of resistance, where increasing the patient dose may be of clinical benefit. Tests may be performed on solid or liquid media and drug concentrations used may vary according to type of the media and method used. The use of binary reporting (sensitive/resistant) of drug susceptibility, whilst useful for programmic treatment does not inform about the degree of resistance. Minimum inhibitory concentrations (MICs) are determined in some research laboratories where the bacilli are cultured over a range of drug concentrations [6]. Variation in methods and the critical concentrations used creates some disparity between laboratories, particularly for strains where the level of resistance is close to the critical concentration for the drug.

Mtb drug resistance is predominantly conferred by the accumulation of mutations (single nucleotide polymorphisms [SNPs], insertions and deletions [indels]) in genes coding for drug-targets or -converting enzymes [7]. To overcome a loss of fitness that arises during the accumulation of such mutations, putative compensatory mechanisms have been described [8–10]. Many mutations conferring drug resistance have been characterized, especially to first-line treatments [11], and their detection offers a means of rapidly assessing susceptibility to anti tuberculosis drugs to improve patient management [11, 12]. However, with the exception of RMP and INH, current molecular tests for resistance lack sensitivity [7]. RMP is a semisynthetic antibiotic that binds to the RNA polymerase β subunit encoded by *rpoB*, inhibiting transcription. Mutations in *rpoB* can cause resistance to RMP [13]. Mutations occur more frequently in an 81 bp region of the gene termed the RMP resistance determining region [14, 15], and contribute to 96 % of resistance phenotypes (predominantly high level), with S450L (*M. tuberculosis* nomenclature) being the most prevalent mutation [16, 17]. It should be noted however that not all mutations result in the same degree of resistance. For example, substitution of histidine with non-polar leucine (H445L) has a much reduced impact compared to the negatively charged aspartate (H445D) (MIC ~2 μg/ml vs. >150 μg/ml) [17]. While cross resistance between RMP and other rifamycins, such as rifabutin and rifapentine, has been recorded [18], the compound structure of the drugs is different. This leads to subtle interaction differences between the binding site and the drugs, and could explain differential mutations causing resistance [19]. Further investigation using similar protein modelling approaches could shed light onto the mechanism of resistance to these drugs and highlight the key residues required for resistance.

INH is a compound that inhibits mycolic acid biosynthesis by binding to an enoyl-acyl carrier protein reductase encoded by the *inhA* gene. It is a pro-drug, which is activated by a catalase-peroxidase enzyme encoded by *katG*. Mutations in *katG* are more tolerated than those in *inhA,* and more frequent in drug sensitive isolates. The *katG* 315 mutations S315N/T account for the majority (60-80 %) of the INH resistance in clinical isolates [20]. Mutations affecting *inhA* usually appear in the promoter region of its operon (denoted *inhA*-promoter), leading to increased transcription. Whilst mutations in *katG* lead to moderate to high levels of resistance (always >1 mg/L), those affecting *inhA* confer a lower level of resistance [20] (<1 mg/L), and therefore if detected could allow INH to play a further role in treatment [21]. Computational initiatives involving protein structure modelling have been applied to understand better the molecular mechanisms of drug resistance, especially where multiple mutations are present. It has been established that the binding affinity of RMP-*rpoB* is most altered by common S450L and H445Y mutants, leading to less effective binding and resistance [22]. Similarly, the S94A mutant leads to decreased affinity of the drug on INH-*inhA* binding, and increased resistance [23].

SM is an aminocyclitol glycoside that binds to 16S rRNA and inhibits protein synthesis. Mutations in the S12 ribosomal protein encoded by *rpsL* have been linked to resistance. These mutations change the tertiary structure of the 16S rRNA leading to decreased affinity to SM and high-level resistance. The majority (54 %) of SM resistance in clinical isolates has been attributed to the K43R mutation in *rpsL* [24]. Whilst mutations in *rpsL* confer a high level of resistance [25], those in *rrs* (encoding 16S rRNA) are thought to contribute to moderate levels of resistance [24, 26], and those in *gidB* confer low levels of resistance [27, 28]. EMB is a first line drug targeting arabinan synthesis, which affects the mycobacterial cell wall. It targets members of the *embCAB* operon, which code for arabinofuranosyl transferases involved in synthesising components of the cell wall. Mutations in *embB*, especially at codons 306, 406 and 497, are frequently observed and give rise to a low level of resistance [29]. The observed range of low to moderate resistance is mutation-specific [30] and thought to differ from other drugs in that it is more a step-wise process, with each mutation increasing the level of resistance [29]. Mutations in *embCAB*, *ubiA,* and *aftA* are thought to accumulate and can cause high levels of resistance observed in some clinical isolates [29].

To improve knowledge of genetic determinants of drug resistance, the use of whole genome association methods has been suggested [31]. Here we undertook whole genome analysis of 144 clinical isolates in the collection of the Special Programme for Research and Training in Tropical Diseases (TDR) [32], for which live material is available to the research community (http://bccm.belspo.be). The isolates were sourced from the TDR strain bank and were selected to encompass diverse geographical settings representing the four major *M. tuberculosis* lineages [33], as well as include susceptible and resistance strains within lineage. Drug susceptibility testing was performed using RMP, INH, EMB, SM, kanamycin (KAN), capreomycin (CAP), ethionamide (ETH), ofloxacin (OFL), and para-aminosalisylic acid (PAS). No testing was performed for pyrazinamide (PZA). The completeness of phenotypic MICs was highest in first-line drugs. A genome-wide association approach was used on 127 isolates to detect genetic variants associated with drug resistance. Typically, failing to account for population structure, in particular the phylogenetic- or lineage-related clustering, potentially involving outbreaks, may lead to false positive associations. Adjusting for principal components and removing lineage-informative mutations in regression analyses have been used to control for these confounding effects. The use of mixed regression models, which include a SNP-based estimate of between sample kinship as a random effect, is considered a more robust approach for isolates that are highly related or with familial relationships [34]. Application of these approaches identified established resistance loci [35]. Many of the loci were supported by evidence of evolutionary convergence, that is, the repeated and independent emergence of mutations in phenotypically resistant strains, identified as homoplastic SNPs in a phylogenetic tree [36].

Mutations in coding regions can have many different effects on a protein structure and function [37–40]. Structural bioinformatics approaches for modelling and mutation analysis were applied to the polymorphisms identified in the *rpoB* and *katG* genes. The effect of mutations on protein stability and interactions was assessed quantitatively with well-established computational methods, shedding light on molecular mechanisms giving rise to observed drug resistance. Whilst second-line drug resistance was tested for only 40 isolates - not sufficient to perform a genome-wide analysis - a number of novel mutations in candidate genes were identified.

## Methods

### Isolates and phenotypic methods

Susceptibility testing was performed in the Antwerp laboratory where the samples were stored as part of the Special Programme for Research and Training in Tropical Diseases (TDR) strain bank [32]. Isolated Mtb strains were previously collected from various geographical sites to create a diverse collection of well characterised drug resistant strains to provide a resource for the TB research community [32]. Single colonies were selected and kept on Löwenstein-Jensen (LJ) culture for drug susceptibility testing. Resistance patterns for the first line drugs were determined using the proportion method, with the critical concentrations 0.2 μg/ml INH, 40 μg/ml RMP, 4 μg/ml SM, and 2 μg/ml EMB. MIC were also investigated on LJ for RMP (10, 20, 30, 40, 80, and 120 μg/ml), INH (0.05, 0.2, 0.8, 1.6, and 3.2 μg/ml), SM (1, 2, 4, 8, and 16 μg/ml), and EMB (1, 2, 4, and 8 μg/ml). The critical thresholds of MIC for calling resistance were 0.2, 2, 4, and 40 μg/ml for INH, EMB, SM, and RMP, respectively [32]. The MIC values were discretised into three groups (susceptible, intermediate, and fully resistant) using natural cut-offs in their empirical distributions.

For the second line drugs PAS was tested on LJ at 0.5 μg/ml. The other drugs were tested on Middlebrook 7H11 agar at the following concentrations: OFL 2 μg/ml, KAN 6 μg/ml, CAP 10 μg/ml, and ETH 10 μg/ml. The proportion method was used for all second line drugs with a critical proportion of 1 %. Lyophilised isolates were sent to the London laboratory where they were grown on LJ prior to DNA extraction using the Bilthoven RFLP methodology [41].

### Sequence data and variant calling

All DNA samples underwent Illumina sequencing on the HiSeq 2000 platform at the KAUST genomic facility, generating paired-end reads of 150 bp (Additional file 1: Table S1, pathogenseq.lshtm.ac.uk/tdr, Additional file 1: Table S2). All raw sequence data can be downloaded from the ENA short read archive (accession number PRJEB11653). For the raw sequence data, *trimmomatic* (v0.33) software [42] (parameters: LEADING:3 TRAILING:3 SLIDINGWINDOW:4:20 MINLEN:36) was used to remove or truncate reads of low quality. High quality reads were then mapped to the H37Rv reference genome (Genbank accession: AL123456.3) using the *BWA-mem* (v0.7.12) algorithm [43] (parameters: -c 100 -M -T 50). From the resulting alignments, *SAMtools* (v1.3) [44] and *GATK* (v3.5) [45] software (default parameter settings) were used to call SNPs and small indels, and the interaction of variants between the methods retained. Mappability values were calculated along the reference genome using *GEM-Mappability* software with a *k-mer* length of 50 bp and a 0.04 % substitution threshold [46]. Non-unique SNP sites (mappability values greater than one) were removed. Sample genotypes were called using the majority allele (minimum frequency 75 %) in positions supported by at least 20-fold total genome coverage, otherwise they were classified as missing. Isolates or SNPs with in excess of 10 % missing genotype calls were excluded. The final dataset included 144 isolates and 17,952 genome-wide SNPs.

### Population structure and association analysis

The best-scoring maximum likelihood phylogenetic tree rooted on *Mycobacterium canetti* was constructed by *RAxML* (v8.2) software [47] (parameters: -T 10 -f a -x 12345 -m GTRGAMMA -p 12345 -N 100) using the 17,952 high quality SNP sites. *M. canetti* is a predecessor of *M. tuberculosis* and therefore provides a convenient root to map for both ancient and modern strains. Spoligotypes were inferred *in silico* using *SpolPred* [48] and matched perfectly with available experimental results. Strain-types were determined using lineage-specific SNPs [33]. Further population structure assessment was performed using principal components analysis [49], leading to covariates for adjustment in association analyses. Logistic regression models were employed to estimate the strength of association between the binary drug resistance outcome (resistance vs. susceptible) and the aggregate number of mutations by coding region, RNA loci, and intergenic regions, as well as operons. Similarly, proportional odds models were applied to a trichotomous phenotype based on MIC values (susceptible, intermediate and full resistance). As expected a number of genes would be reported as significant due to a large amount of cross-resistance between drugs, and we adjusted for the presence of other resistance in the regression models. The main association analysis using mixed models with a SNP inferred kinship matrix as a random effect was implemented in *EMMA* (v.1.1.2) [34]. The operons or functional units containing clusters of genes under the control of the same promoter were determined from *TBDB* [50]. Gene function was extracted from *Tuberculist* [51]. Permutation tests based on resampling MIC values were performed to establish a statistical significance cut-off for each drug to account for false positives arising from multiple locus tests. The established cut-offs were RMP 1.58 × 10^-5^, INH 1.67 × 10^-5^_,_ SM 2.73 × 10^-5^, and EMB 1.77 × 10^-5^. All statistical analyses were performed using *R* (v3.2) software. To identify SNPs enriched by convergent evolution, the *phyC* approach [36] was employed using an available implementation [52].

### Protein mutation modelling

An *apo* crystal structure for *katG* (1SJ2 [53]) was available and downloaded from the Protein Data Bank (*PDBe* [54]). A protein homology model for *rpoB* was obtained from the Chopin database (http://mordred.bioc.cam.ac.uk/chopin). Reliable models could not be found or generated for *embB*, *rpsL* or other loci identified in our work. Structures of the drug compounds INH and RMP where obtained from the chemical components section of PDBe and used in *Autodock vina* [55] to perform *in silico* drug docking. The *mCSM (*http://structure.bioc.cam.ac.uk/mcsm*)* and *DUET (*http://structure.bioc.cam.ac.uk/duet*)* web servers were used to assess changes in protein stability and mCSM-PPI (http://bleoberis.bioc.cam.ac.uk/mcsm/protein_protein) to quantify effects on protein-protein interactions [56, 57].

## Results

### Genetic polymorphisms

The 144 isolates represented a broad global distribution, sourced from 24 countries in four continents (Additional file 2: Figure S1, Additional file 1: Table S1). All the African isolates were lineage 4 strains, and only Asia contributed lineage 1 strains. Across the isolates, 19,248 SNP sites were identified, including 17,092 (89 %) in coding regions of the genome (11,003 [(57 %] non-synonymous mutations). The SNP allele frequency spectrum revealed, as expected, the majority of variants were rare (12,244 [63.5 %] SNPs present in only one isolate; Additional file 3: Figure S2). Both a phylogenetic tree and a principal component analysis based on the ~19 k SNPs showed congruent clustering by lineage (Additional file 4: Figure S3). The tree revealed a cluster of nine Rwandan strains, which were separated by low numbers of SNP differences (range 1-17 SNPs), implying potential transmission. It also revealed one sample reported as susceptible to EMB was likely to be resistant due to its location on the tree within a cluster of isolates with resistance.

### Drug resistance

**Fig. 1:**
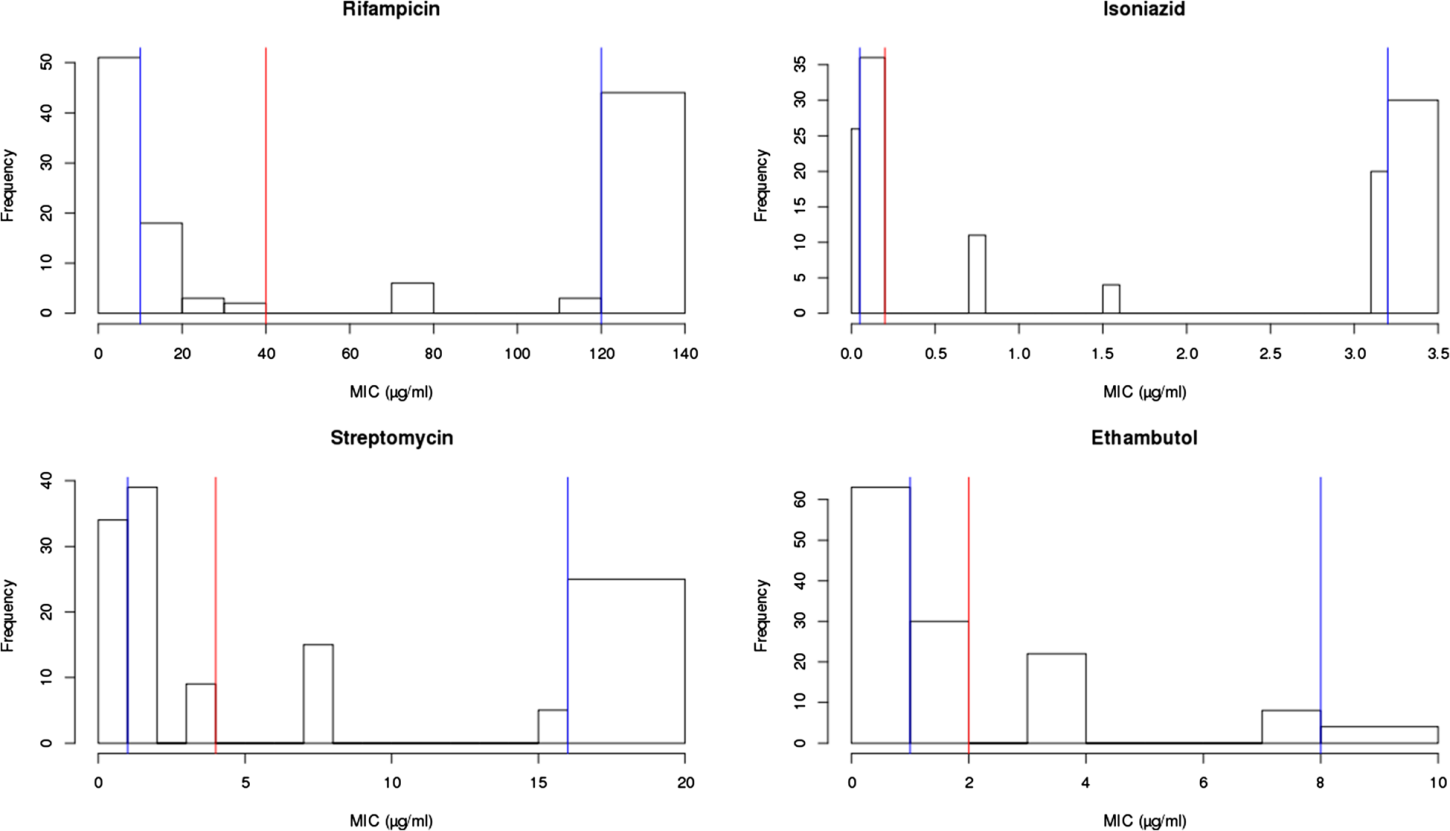
The distribution of MIC values for rifampicin, isoniazid, streptomycin, and ethambutol. The *red vertical line* is the standard susceptible-resistance threshold (rifampicin 40 μg/ml, isoniazid 0.2 μg/ml, streptomycin 4 μg/ml, ethambutol 2 μg/ml). The two *blue vertical lines* define the three levels (susceptible, intermediate and full resistance): rifampicin (10, 120 μg/ml), isoniazid (0.05, 3.2 μg/ml), streptomycin (1, 16 μg/ml) and ethambutol (1, 8 μg/ml). *MIC* minimum inhibitory concentrations

The drug susceptibility test MIC values for the four first line drugs were available for 144 isolates, and 17 strains were removed due to poor sequence coverage and quality. For the remaining 127 isolates, similar numbers of sensitive and resistant strains were present (Fig. 1). For the trichotomised MIC values, the intermediate resistance group comprised less than 20 % of isolates across drugs (see Fig. 1 for breakpoints). There was a high correlation between INH and other drug MIC values (Spearman’s *rho* >0.31, p <0.006), and in total there were 14 distinct drug resistance combinations across the four first-line drugs, in keeping with the step-wise and combination nature of therapies. Twelve (9.4 %) isolates were pan-resistant, 38 (29.9 %) pan-susceptible, and 42 (33.1 %) multi-drug resistant (using dichotomised values, Additional file 1: Table S3). The *TB profiler* [11] was used to infer drug resistance profiles *in silico* from known drug resistance mutations. Assuming the drug susceptibility tests as the reference standard, the computationally inferred resistance profiles were highly accurate for RMP (sensitivity/specificity: 0.962/1.000) and INH (0.908/0.935), suggesting the sequencing result would be of clinical value for detecting MDR-TB. The performance for SM (sensitivity/specificity: 0.511/0.960) and EMB (0.971/0.839) was less accurate. High predictive values will be needed to guide the use of SM and EMB in patients with MDR and XDR-TB. It would appear that the repertories of mutations and loci for these drugs still need to be elucidated and that intermediate resistance with MIC values close to the resistance cut-offs could pose problems using binary outcome values when correlating genotype and phenotype. Mutations in the *gid* gene are not included in *TB Profiler* as they cause only intermediate levels of SM resistance. We observed twenty *gid* markers and their incorporation increased the SM sensitivity to 82 %. Further, it was predicted that 14 (11 %) isolates were likely to be PZA resistance. In particular, each of the 14 isolates had at least one known drug resistance conferring mutation in the *pncA* gene (Ala171Pro, Arg121Pro, Asp8Ala, Gln10Pro, His57Pro, His82Asp, Ile31Ser, Ser66Pro, Thr76Pro [n = 2], Trp68Ser, Tyr103His, and Val125Gly [n = 2]).

In an attempt to search for new mutations involved in drug resistance a genome wide association analysis was performed on both trichotomous MIC and binary resistance phenotypes. Both single SNP and locus-wide association testing were considered. Similar to a rare variant analysis, the number of (non-synonymous) mutations per sample, per gene and operon was calculated, and correlated with the phenotype. In addition to association analysis, the complementary *phyC* approach was applied. This approach aims to identify loci under convergent evolution in resistance branches of the tree. A summary of all statistically significant results is presented (Table 1), and we focus on each drug separately.

**Table 1:** First-line drug related SNPs identified in association and convergent evolution analysis

Drug	Gene	SNP mutations (% in resistant isolates)
Rifampicin	*rpoB*	T400A (3.8), D435V (9.4), H445D/Y (11.3),
		H445R (5.7), **S450W/L** (60.4), I491V/F (3.8)
Isoniazid	*katG*	**S315N** (69.2)
Isoniazid	*Rv1482c-fabG1 (inhA*-promoter)	**C-15 T** (24.6)
Streptomycin	*rpsL*	**K43R** (24.4)
Ethambutol	*embB*	C12T (5.9), M306I (14.7*), **M306V** (17.7*), D354A (11.8), G406S/C (11.8), G406D/A (11.8**), **Q497P/R** (17.7***), D1024N (8.8)
Ethambutol	*cadI*	**C-39 T** (8.8)

The genes were identified using aggregated mutation mixed models. The SNPs were identified using the *phyC* method and those also found using the GWAS mixed model approach are highlighted in **bold**

*SNP* single nucleotide polymorphism, *GWAS* genome-wide association study

*observed in “sensitive” strains at frequency 3.2 %; **4.3 %; ***1.1 %; all P < 1 × 10^-5^ from association analysis

#### Rifampicin

**Fig. 2:**
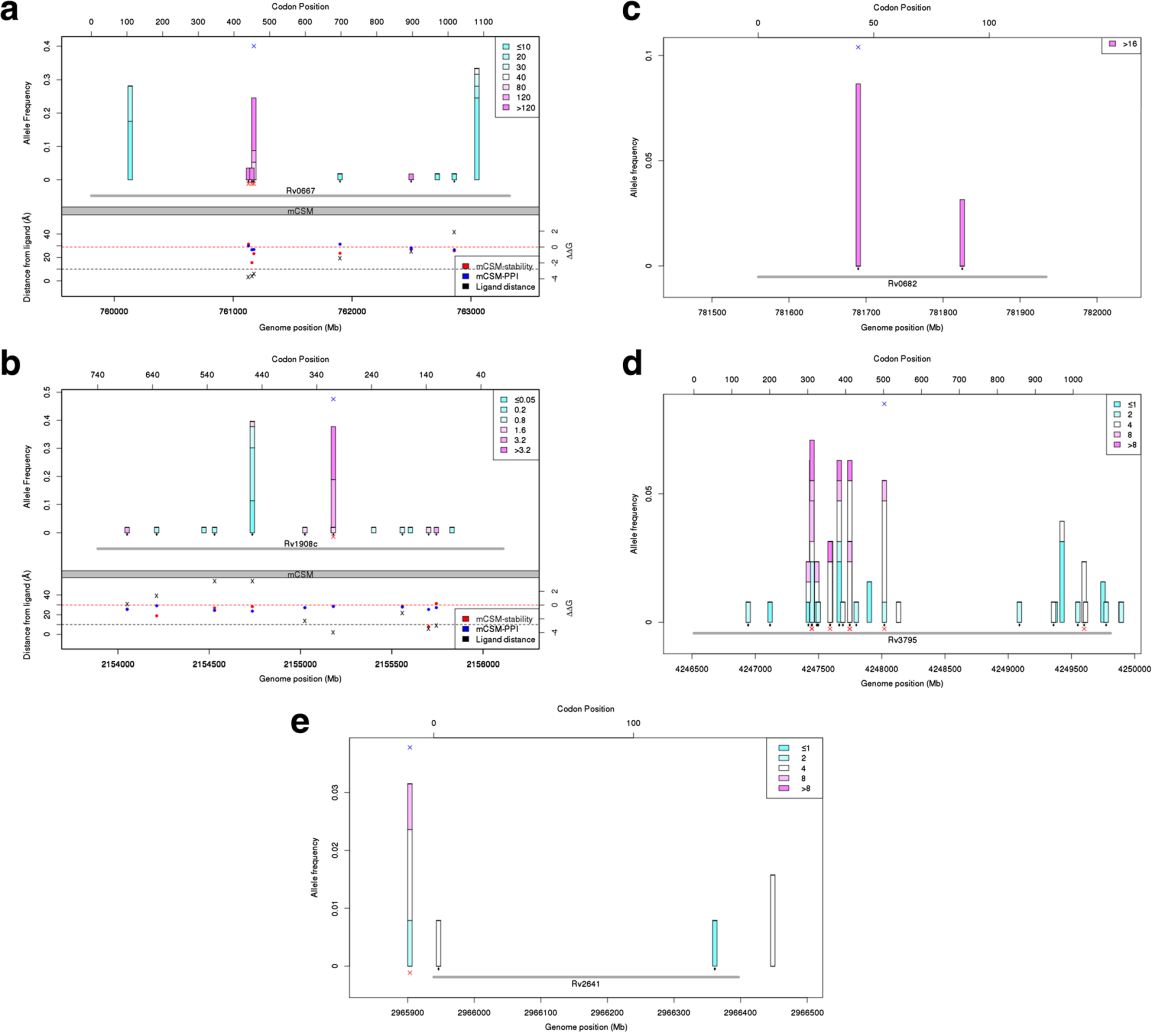
SNPs in candidate genes in isolates with a single mutation in each locus. The *bars* represent the allele frequency of the SNPs, and are coloured according to the MIC value. *Black dots* under bars represent non-synonymous mutations. *Blue* and *red crosses* represent mutations that have been found to be significant in the association and the convergent evolution *phyC* analyses, respectively. Structural data are available only for *rpoB* and *katG* (*bottom panels*). The protein stability and protein-protein interaction changes induced by the SNP as calculated by *mCSM* software are represented by the *red* and *blue points*, respectively, and magnitude is represented on the *right y-axis*. The distance of each mutated codon from the docked drug *(left y-axis*) is denoted by the *black crosses*. **a** Rv0667 *rpoB* (rifampicin). **b** Rv1908c *katG* (isoniazid). **c** Rv0682 *rpsL* (streptomycin). **d** Rv3795 *embB* (ethambutol). **e** Rv2641 *cadI* (ethambutol). *SNPs* single nucleotide polymorphisms, *MIC* minimum inhibitory concentration

Genome-wide analysis using both binary trait or MIC values revealed, as expected, that the *rpoB* gene (p <1 × 10^-20^) and its operon (p <1 × 10^-10^) were associated with RMP resistance. One tri-allelic SNP in *rpoB* at position 761,155 (codon 450: S450L 30/127, S450W 2/127) was associated with the majority of RMP drug resistance (60 %). There were six significant SNPs under convergent evolution (p <0.05) in *rpoB* (codons 450, 445 (*x*2), 435, 400, and 491), one in *rpoC* (N416S mutation, two isolates, a known compensatory mechanism) and one in *lldD2* (codon 2 synonymous, 16 isolates). Fifty isolates (93 % of RMP resistant strains) had at least one mutation in the *rpoB* gene in the RMP resistance determining region (codon range 400-491) (Fig. 2a). Three isolates had two mutations in this region. Two isolates had mutations in codons 400 and 450 and one strain had mutations in codons 450 and 491. All except four isolates with a mutation in *rpoB* had MIC values of at least 120 μg/ml and the remaining four had values of 80 μg/ml.

#### Isoniazid

The association analysis revealed the *Rv1907c-furA* operon (p <1 × 10^-13^), which contains the *katG* gene (p <1 × 10^-9^) as the most significant association (Fig. 2b). Other loci identified included the *fabG1-hemZ* operon (contains the *inhA* gene and promoter). Using MIC values, the *Rv1907c-furA* (p <2 × 10^-5^) operon and *katG* and *Rv1979c* genes were found to be associated with INH resistance. A SNP-based GWAS revealed a single polymorphism association in *katG* (position 2,155,168, S315T/N, P <4.33 × 10^-18^). This SNP was supported by *phyC* analysis, which also revealed another site under convergent evolution in *inhA* promoter. Overall, 47 (75 % of INH resistant) strains have a SNP in position 2,155,168 (S315T 41 isolates, S315N four strains), of which 43 have an MIC value of at least 3.2 μg/ml, while the remaining two had values of 0.8 and 1.6 μg/ml. Twenty-one isolates have a SNP in the *fabG1-hemZ* operon, with MIC values ranging from 0.8 to ≥3.2 μg/ml. Of the 16 isolates that only have one SNP in the *fabG1-hemZ* operon, half had MIC values in excess of 0.8 μg/ml. The three isolates with mutations at both the *fabG1* promoter and *inhA* had an MIC value in excess of 1.6 μg/ml. Three (of six) isolates with a mutation in the promoter and an MIC of at least 3.2 μg/ml also have the *katG* S315T mutation. One mutation in the *katG* promoter region was found in a drug sensitive sample.

#### Streptomycin

The association analysis identified the *rpsL-rpsG* operon and the *rpsL* gene as being associated with SM resistance (Fig. 2c). The *rpsL* locus was also found by analysing MIC values, and a SNP-based approach identified one mutation (position 781,687, K43R, 11 isolates, 26 % of resistant strains) within the gene. The *phyC* method identified two SNPs in the rRNA gene *rrs* (514 A- > C, four isolates; 517 C- > T, three strains). All isolates, except one, had an MIC of greater than 16 μg/ml. One sample with the 1,472,362 C- > T mutation had an MIC of 8 μg/ml.

#### Ethambutol

A binary phenotype analysis identified the *embA-embB* operon (p <1 × 10^-10^) and the *embB* gene (p <1 × 10^-13^) (Fig. 2d). This result was confirmed in an analysis of the MIC phenotype (operon p <1 × 10^-10^, gene p <1 × 10^-8^). A SNP-based association analysis revealed one in the *embB* gene (position 4,248,003) and one in the promoter of *cadI,* where the latter was also found using the *phyC* method (four isolates) (Fig. 2e) The *phyC* approach identified seven SNPs in *embB* (codons 306 [*x*2, 22 isolates], 354 [four resistant isolates], 406 [*x*2, 12 isolates], 497 [seven isolates], and 1024 [two isolates]). Three isolates had mutations in two of these positions and all others had only one mutation. There was a great range of MIC values in isolates containing these mutations with some codons having both sensitive and resistant strains. For example, 6/22 isolates with mutations in codon 306 had MIC values of at most 2 μg/ml. Mutations in the *embA* promoter were also present, but not found to have a consistent effect on the MIC values when combined with mutations in *embB*. The additive effect of mutations in the candidate genes *embB*, *embA*, *embA* promoter, *embC*, *embR*, and *ubiA* correlated modestly with MIC values (*rho* = 0.24, Additional file 5: Figure S4). The aggregated mutation approach revealed that the *pncA* gene may be associated with EMB resistance, but this was most likely due to cross-resistance from the predicted PZA resistant cases (n = 14).

#### Use of MIC values

The correlation between association p-values using binary resistance (susceptible, resistant) and trichotomous MIC was modest (RMP 0.386, INH 0.311, EMB 0.309, and SM 0.360), but led to near identical strongest hits. However, there were some discrepancies in the findings for EMB and SM. The majority of isolates (11/15) that were EMB phenotypically susceptible, but with known drug resistance mutations, had an MIC value of 2 μg/ml. This value is on the upper bound of the sensitive range, but low-level resistance may be predicted as they had known EMB drug resistance mutations. The majority of SM false negative (15/22) isolates had an MIC value of 8 μg/ml, which is on the lower limit of the resistance cut-off. Mutations in *gid* are known to cause low levels of resistance, and the majority (19/22) of false negative strains contained mutations in that gene. The additive effect of mutations in both EMB and SM candidate genes correlated with increasing MIC value (EMB: *rho* = 0.24, slope = 0.29, p = 0.003; SM: *rho* = 0.48, slope = 3.59, p = 1.65 × 10^-8^; Additional file 6: Figure S5), and may provide some evidence of accumulating low resistance mutations.

An exciting prospect is the use of MIC values to infer the additive and interaction effects of each mutation. Unfortunately, the relatively small sample size did not allow a rigorous statistical approach to look for interactions. However, the frequencies of combinations of mutations for RMP, INH, EMB, and SM, and their MIC values are presented (Additional file 1: Table S4). Using these data, statistical models were fitted with all mutations included, to allow an assessment of the MIC variation explained and their independent effects in the presence of others (Additional file 6: Figure S5). For RMP and INH, a high proportion of MIC variation is explained by single mutations (RMP: *rpoB* 450, 48.4 %, INH: *katG* 315, 73.8 %). However, for EMB and SM, single mutations explained at most ~30 % (SM: *rpsL* codon 43 – 32.4 %, EMB: *embB* codon 306 – 30.0 %), with the largest proportion due to unknown factors (SM: 44.0 %, EMB: 37.4 %). This analysis further supports that other variants need to be identified for EMB and SM drugs.

We compared the association results from the mixed models using all available data to regression-based approaches that adjusted for the principal components (explained ~60 % of variation) and removed 414 lineage- and clade-specific markers and eight highly related Rwandan strains (Additional file 4: Figure S3). There was a moderate level of correlation between the approaches for all outcomes (minimum *rho* - RMP: 0.66, INH: 0.54, SM: 0.20, EMB: 0.34). This correlation translated into identical top hits for association (Table 1), except for the *cadI* gene, which was identified only by the mixed model approach at the stringent significance cut-off. CadI is a protein that can be induced by cadmium, and is thought to possess similar functions to the metallothioneins and protects the bacterium against metal toxicity (http://tuberculist.epfl.ch).

#### Second-line drugs

Forty-four (35.8 %) isolates were tested for second line drug resistance, and the polymorphism in known candidates was considered (Table 2). Of the six isolates that were resistant to PAS, mutations at candidate genes (*folC, ribD, thyA*, and *thyX*) were observed in all isolates (*folC* E40G, I43G, D135G; *thyA* Y94C, Q97R, V135F; and *thyX* promoter G-16A (n = 2), T-43G). Seven isolates had ETH resistance, of which all had mutations in drug resistance candidate genes (*ethA* R469P, n = 1; *ethR-fabG1* promoter region C-15 T, n = 6; and *inhA* gene S94, n = 1). Three isolates had resistance to OFL, with known mutations in the *gyrA* gene (D94G, n = 2; N499D, n = 1). Two isolates had resistance to CAP, with unreported mutations in candidate genes (*rrs* A1205G, n = 1; *tlyA* gene G196E, n = 1). No indels were identified in these genes.

**Table 2:** Second-line drug related mutations in candidate genes

Drug	No. resistant	Locus (codon [no. isolates])
Para-aminosalisylic acid	6	*folC* (E40G[1], I43G[1], D135G[1]);
		*thyA (* **Y94C**[1], Q97R[1], **V135F**[1]);
		*thyX* promoter (**G16A** [2], **T43G** [1]).
Ethionamide	7	*ethA* (**R469P**[1]);
		*ethR-fabG1* promoter (C15T[6]);
		*inhA* (S94[1])
Ofloxacin	3	*gyrA (*D94G [2]; N499D [1]).
Capreomycin	2	*rrs* (**A1205G**[1]); *tlyA* (**G196E** [1])

Previously unreported in **bold**

#### Effects on protein structure and function

The availability of structural information for *katG* and *rpoB* genes allowed us to assess the potential functional effects of the mutations identified and their ability to predict drug resistance. The respective INH and RMP drugs were computationally docked into the models, delimiting the residues of the drug binding site. The *mCSM* and *DUET* servers were used to quantify the influence of mutations on protein stability and protein-protein interactions (measured by the change in Gibbs free energy ΔΔG between the wild-type and mutant structures). These factors, individually or combined could lead to drug resistance. The predictions obtained are summarized in Additional file 1: Table S5.

**Fig. 3:**
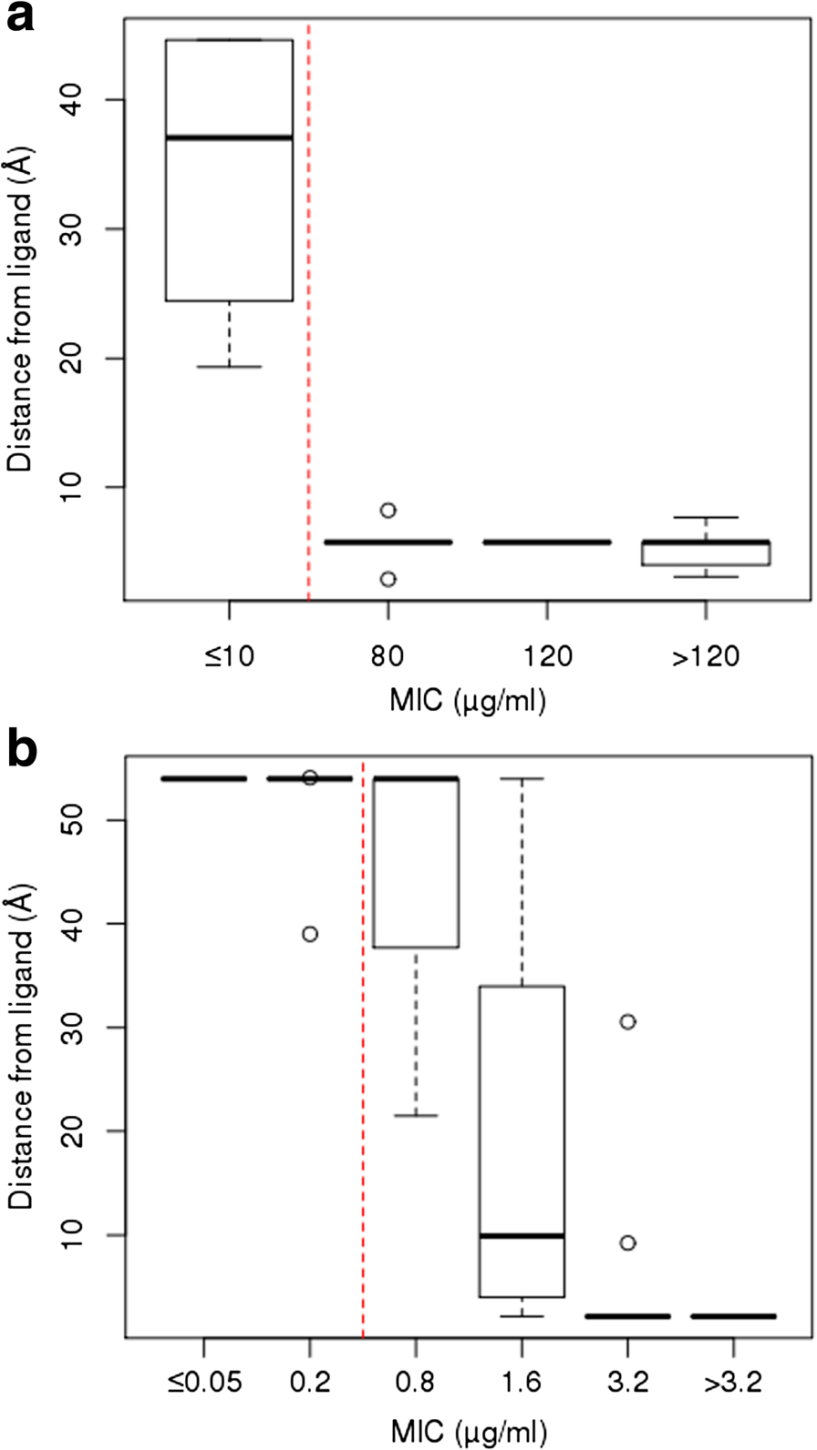
Boxplot showing the distributions of the distance of the mutated codon to the drug for all the SNPs in each MIC level in (**a**) RMP-*rpoB* and (**b**) INH-*katG. Vertical red line* is the resistance cut-off. *SNP* single nucleotide polymorphism, *MIC* minimum inhibitory concentration, *RMP* rifampicin, *INH* isoniazid

**Fig. 4:**
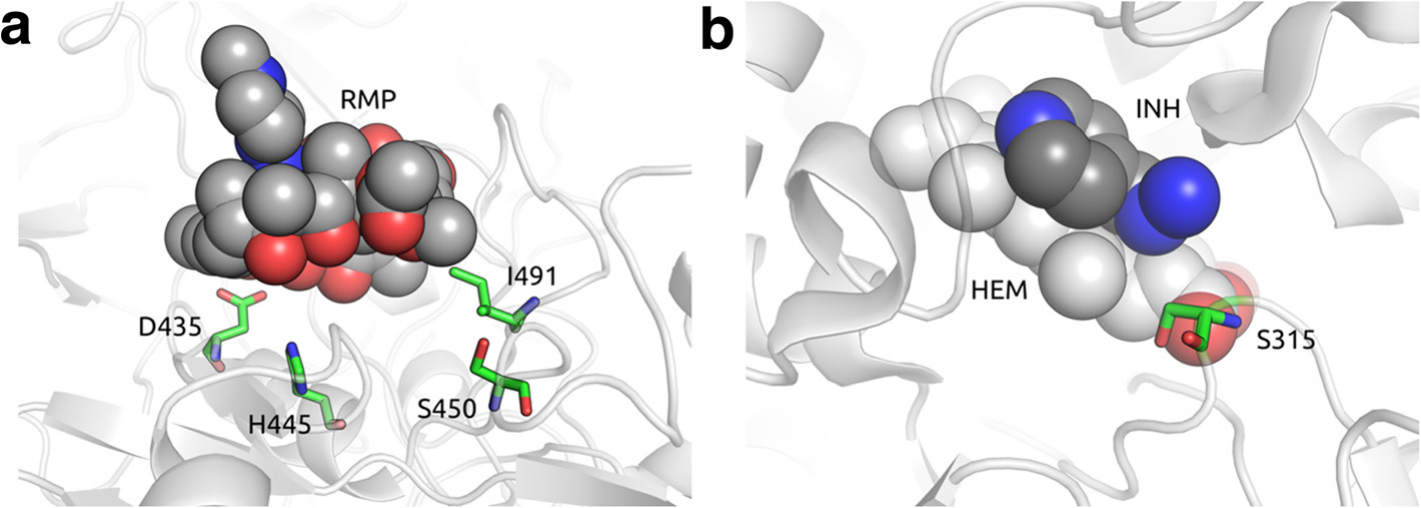
Mutations in binding site regions. **a** depicts the spatial distribution of mutated residues in the *rpoB*-RMP complex while (**b**) shows the residue Ser315 in *katG*-INH complex (residues depicted with carbons in green). The distance between the residues and the ligands (depicted with carbons in dark grey) vary from 2.1 to 5.7 Å. *RMP* rifampicin, *INH* isoniazid

Across the eleven RMP resistance codons analysed in *rpoB* and ten INH resistance codons of *katG*, no strong correlation of the changes in protein stability with the proportion of drug resistant isolates with each mutation was observed (*rho* < 0.05, p >0.05). There was weak evidence that drug resistant isolates had mutations that were more destabilizing (p <0.10). The mutations in *katG* were not located near the homodimer interface, while further structural information is necessary to characterise the *rpoB* interactions. However, across both drugs there was a strong association between (a shorter) distance of the mutation to the ligand in the protein structure and resistance (greater MIC values) (*rpoB rho* = -0.79, p = 8.1 × 10^-6^; *katG rho* = -0.72, p = 0.0012) (Fig. 3). For RMP, isolates with MIC values of at least 80 μg/ml had mutations located close to the drug binding site (median distance of 5.77 Å, all values less than 10 Å) as depicted in Fig. 4, compared to isolates with MIC values of ≤10 μg/ml (median distance of 37.08 Å). For INH, isolates with MIC resistance values of at least 3.2 μg/ml had mutations directly interacting with the drug (median 2.15 Å) (Fig. 4), whilst isolates with intermediate resistance (1.6 μg/ml) mutations located further away (median 9.93 Å), and mutations in susceptible strains (MIC values less than 0.8 μg/ml) were even more remote (median 53.97 Å). Additional file 7: Figure S6 shows the molecular interactions established by mutated residues in *katG* and *rpoB*, with most of the effects of mutations influencing interactions established directly with the drug molecule, by destabilizing the surrounding region via loss of interactions or the introduction of steric clashes. Whether we can predict the resistance of a mutation using its distance to a ligand site will have to be verified using other protein structure models, when they become available.

## Discussion

Early characterisation of drug resistance mutations would assist TB patient management and avoid treating individuals with inefficacious toxic regimens [11]. Current testing for resistance to most anti-tuberculosis drugs, as applied to isolates in TDR, involves isolation and culture of the bacteria followed by exposure to the drug, a process that takes weeks or months [11]. However, the direct sequencing of *M. tuberculosis* from sputum from suspected drug resistant patients [58] and the development of rapid strain profiling tools, suggests that culture-free approaches have a role in the management of TB [11]. For some drugs, such as RMP and INH, resistant mutations are well characterised, but for others such as SM, EMB and second-line treatments, existing databases lack specificity and sensitivity [11]. We performed a genome-wide association approach on SM and first-line treatments and assessed its ability to confirm existing, and identify new, variants that cause drug resistance. Whilst genome-wide association methods have become established for disease susceptibility studies in humans, their application in pathogens is still in its infancy [31]. Population structure can confound analyses and lead to false positive results. For TB, widespread drug resistance may be over represented in particular lineages or clades, causing lineage specific SNPs that confound analyses. This confounding was handled by a mixed model, but alternative approaches were considered, in particular, removal of all lineage- and clade-specific markers or inclusion of principal components as surrogates for lineages within the regression model. These approaches led to near identical top association hits, in part reflecting the strong signal of the resistance-related mutations across clades, the dominant clustering of discrete lineages in the phylogeny, and the modest number of highly related or outbreak-based isolates (e.g. Rwandan strains). Our work suggested that the use of kinship matrices within mixed models may avoid the removal of lineage-informative SNPs and highly related strains, especially those involved in an outbreak or transmission study. This observation is supported in human GWAS studies with familial relationships, where mixed models have been found to be more robust to false positive associations than principal components adjustment [59].

A limitation of the study was the representation of geographic origins and lineages, as we were restricted by availability of strains collected for this extremely well characterized collection. A second limitation was the small sample size, especially for analyses of second-line drugs, where a genome-wide approach could not be implemented. However, where sample sizes were sufficient our genome-wide analysis reported genes known to be involved in first-line RMP, INH, SM, and EMB drug resistance. The use of MIC values has been advocated as a more sensitive measure, but the potential lack of a symmetric distribution of values (as shown in our data) could lead to invalidation of assumptions for linear models. We took the pragmatic approach of discretising the values into three natural groups (resistant, sensitive, and intermediate) allowing an alternative modelling strategy (proportional odds model) to be employed. The correlation between association analysis p-values using both binary and trichotomised MIC values was modest (range: 0.31-0.39). Some isolates with intermediate SM resistance had no known drug resistance mutations in *rpsL* and *rrs*, and even after inclusion of *gid* mutations, additional causal mutations or genes to explain phenotypic variation remained unidentified. Larger sample sizes would facilitate the use of raw MIC values and therefore advance the detection of variants that confer intermediate resistance. Many of the results were also confirmed using convergent evolution methods, which require smaller sample sizes than genome-wide approaches, and should prove to be a powerful and robust method to detect drug resistance mutations in *M. tuberculosis*, and possibly other pathogens. There are a number of isolates that have very high levels of resistance to both EMB and SM but do not present any mutations in known candidate genes. It is evident that there are rare SNPs occurring in unknown genes that confer EMB resistance. Similarly, there are many isolates with more than one mutation in candidate genes and high levels of susceptibility. Not all mutations in these genes will have an effect on resistance levels, and interactions between the drug and its target should be considered.

The use of protein structures determined by X-ray crystallography or as homology can provide extra validation and an insight into the mechanism of drug resistance conferred by mutations. It has been shown that mutations in the RMP binding site can cause resistance due to disturbance of the active site both in Mtb and in other bacteria [22]. An exciting finding was the strong correlation between the MIC values and the distance in the three-dimensional structure of the mutated residue to the drug docking ligand. This observation seems novel to Mtb. If it holds for other genes as their protein structures become available, then potential drug resistance mutations could be predicted *in silico* in a genome-wide screen. The binding sites of the rifamycins have been shown to be in similar locations and these observations would be expected to be similar for closely related drugs [60]. It could also provide a future high throughput way of integrating genomic and protein structure data to make predictions about drug resistance mutations. In particular, rare SNPs with low allele frequencies may not be detected in association analyses; however, prediction of the distance of the mutated codon to a ligand or its effect on overall stability or protein-protein interactions can provide a complementary approach to identify new drug resistance conferring mutations. Indeed, variants such as the *rpoB* V170F mutant are present in only one isolate in our dataset but it was flagged up as an interesting SNP due to its proximity to the docked RMP ligand in the homology model. This *rpoB* SNP has been attributed to drug resistance by earlier studies^12^.

## Conclusions

Overall, our work has demonstrated the potential of the genome-wide association and selection approaches to identify mutations and genes associated with resistance. We have also shown that if protein structures are available, then the effects of mutations in genes on resistance may be predicted *in silico*. This could facilitate the prediction of the effects of mutations on novel drugs and potential resistance. Ultimately, such insights will assist with patient treatment and management, and disease control.

## Availability of data and materials

All raw sequence data can be downloaded from the ENA short read archive (accession number PRJEB11653).

## Additional files

Additional file 1: Table S1. The isolates according to geographic location and phenotypic drug resistance. CAR Central African Republic; DRC Democratic Republic of Congo, L1-L4 lineages 1 to 4, (first line drugs) RMP = rifampicin, INH = isoniazid, SM = streptomycin, EMB = ethambutol; (second line drugs) OFL = ofloxacin, KAN = kanamycin, CAP = capreomycin, Et = ethionamide, P = Para-aminosalisylic acid. **Table S2**. The isolate ENA accession numbers and MIC values. RMP rifampicin, INH isoniazid, SM streptomycin, EMB ethambutol. **Table S3**. Drug susceptibility profiles for rifampicin, isoniazid, streptomycin and ethambutol. R = resistance, S = sensitive; 13 different profiles were identified across 127 independent isolates; Multi-drug resistant in italics. **Table S4**. Combinations of mutations and their frequency (N) in drug resistance candidate genes. a) Rifampicin. b) Isoniazid. c) Streptomycin. d) Ethambutol. * single mutation, ** double mutations, *** triple mutations; SNP mutations in a single sample have been aggregated into a “rare” column. **Table S5**. Predicted effects of mutations. (DOCX 55 kb) Additional file 2: Figure S1. The global distribution of geographic origin and lineage of the isolates. Lineages one to four are represented by blue, green, purple, and red, respectively. (PNG 265 kb) Additional file 3: Figure S2. SNP allele frequency spectrum. A large number of rare variants are observed. Peaks with higher allele frequency reflect the presence of lineage and sub-lineage specific SNPs. (PNG 33 kb) Additional file 4: Figure S3. Population structure analysis of the 144 isolates show clustering by lineage (Lineages one to four are represented by blue, green, purple, and red points, respectively). (a) A phylogenetic tree rooted with *M. canetti*. (b) First two principal components represent 33 % and 30.5 % of the variation explained between isolates, respectively. (ZIP 105 kb) Additional file 5: Figure S4. The relationship between the total number of non-synonymous SNPs in candidate loci and the MIC values. The size of the circle represents the number of isolates. a) Ethambutol (*embB, embA, embA promoter, embC, embR* and *ubiA*). b) Streptomycin (*rpsL, rrs*). The size of the circles is proportional to the frequency. The MIC values tend to increase with the number of non-synonymous mutations (ethambutol: *rho* = 0.24, slope = 0.29, p = 0.003; streptomycin: *rho* = 0.48, slope = 3.59, p = 1.65 × 10^-8^). The horizontal blue lines refer to the resistance cut-offs. (ZIP 92 kb) Additional file 6: Figure S5. Percentage of the variation in MIC values explained by each mutated codon in candidate genes. Bars in red represent significant independent associations with increased MIC (p < 0.05). a) Rifampicin. b) Isoniazid. c) Streptomycin. d) Ethambutol. (ZIP 231 kb) Additional file 7: Figure S6. Molecular interactions established by wild-type residues in *katG* and *rpoB* residues. (A) The interactions established by Ser315 in *katG*. Given the proximity of the residue to the ligands INH and HEM, mutations to Asn and Thr, with slightly larger side chains, would potentially cause steric clashes. (B) The interactions of Asp435 in *rpoB*. It directly interacts with RMP via polar interactions that would be disrupted by mutations to Val. (C) Thr400 in *rpoB* is at the end of an alpha helix establishing intra molecular interactions. Giving its distance to RMP, it would be expected that its mutation to Ala would be a lower impact, which would arise from alosteric changes. (D) Ser450 establishes strong intra molecular interactions in the RMP binding site. Mutations to larger residues (Trp and Leu) could disrupt the packing of the region and therefore binding. (E). Ile491 performs hydrophobic interactions with RMP and its neighbouring residues. Mutations to Phe or Val would compromise packing, either inducing steric clashes or compromising packing. (F). His445 performs strong intra molecular interactions, including a donor-pi (*blue dashes*) and hydrogen bond (*red dashes*). Mutations to residues Asp, Tyr or Arg would imply in the loss of the pi interaction as well as potential introduction of steric clashes. (PNG 749 kb)
