# Supplementary material for: Mycobacterium tuberculosis whole genome sequencing and protein structure modelling provides insights into anti-tuberculosis drug resistance
Source: BMC Med. 2016 Mar 23;14:31. doi: 10.1186/s12916-016-0575-9 (PMC4804620; doi:10.1186/s12916-016-0575-9)
Supplement: Additional file 1: Table S1. — The isolates according to geographic location and phenotypic drug resistance. CAR Central African Republic; DRC Democratic Republic of Congo, L1-L4 lineages 1 to 4, (first line drugs) RMP = rifampicin, INH = isoniazid, SM = streptomycin, EMB = ethambutol; (second line drugs) OFL = ofloxacin, KAN = kanamycin, CAP = capreomycin, Et = ethionamide, P = Para-aminosalisylic acid. Table S2. The isolate ENA accession numbers and MIC values. RMP rifampicin, INH isoniazid, SM streptomycin, EMB ethambutol. Table S3. Drug susceptibility profiles for rifampicin, isoniazid, streptomycin and ethambutol. R = resistance, S = sensitive; 13 different profiles were identified across 127 independent isolates; Multi-drug resistant in italics. Table S4. Combinations of mutations and their frequency (N) in drug resistance candidate genes. a) Rifampicin. b) Isoniazid. c) Streptomycin. d) Ethambutol. * single mutation, ** double mutations, *** triple mutations; SNP mutations in a single sample have been aggregated into a “rare” column. Table S5. Predicted effects of mutations. (DOCX 55 kb) [file 12916_2016_575_MOESM1_ESM.docx]

**Table S1**

The samples according to geographic location and phenotypic drug resistance

|  |  | **Number of isolates belonging to lineage** | | | | **Number of isolates showing resistance to** | | | | | | | | |
| --- | --- | --- | --- | --- | --- | --- | --- | --- | --- | --- | --- | --- | --- | --- |
| Source | **Tot.** | **L1** | **L2** | **L3** | **L4** | **SM** | **INH** | **RMP** | **EMB** | **OFL** | **KAN** | **CAP** | **Et** | **PZA** |
| ***Asia*** |  |  |  |  |  |  |  |  |  |  |  |  |  |  |
| Bangladesh | 8 | 4 | 1 | 1 | 2 | 2 | 4 | 1 | 4 | 1 | - | - | 1 | - |
| China (Tibet) | 1 | 1 | - | - | - | 1 | 1 | 1 | - | - | - | - | - | - |
| Nepal | 4 | 1 | 2 | - | 1 | 4 | 3 | 2 | 1 | 1 | - | - | - | 2 |
| Pakistan | 1 | - | - | 1 | - | - | - | - | - | - | - | - | - | - |
| Philippines | 4 | 4 | - | - | - | 1 | 2 | 2 | 1 | - | - | - | 2 | - |
| Sth Korea | 39 | - | 23 | 1 | 15 | 17 | 26 | 17 | 15 | - | - | 1 | 1 | 3 |
| Thailand | 1 | - | 1 | - | - | - | - | - | - | 1 | 1 | 1 | - | - |
|  |  |  |  |  |  |  |  |  |  |  |  |  |  |  |
| ***Africa*** |  |  |  |  |  |  |  |  |  |  |  |  |  |  |
| Cameroon | 1 | - | - | - | 1 | 1 | - | - | - | - | - | - | - | - |
| CAR | 1 | - | - | - | 1 | - | - | - | - | 1 | - | - | - | - |
| Guinea | 1 | - | - | - | 1 | - | 1 | - | - | - | - | - | - | - |
| Guinea Eq. | 1 | - | - | - | 1 | - | 1 | 1 | - | - | - | - | 1 | - |
| Morocco | 4 | - | - | - | 4 | 2 | 3 | 1 | 1 | - | - | - | - | - |
| Niger | 1 | - | - | - | 1 | - | - | - | - | - | 1 | 1 | - | - |
| Nigeria | 2 | - | - | - | 2 | - | 1 | 1 | - | 1 | - | - | 1 | - |
| RDC | 4 | - | - | - | 4 | - | - | - | - | - | 1 | 1 | - | - |
| Rwanda | 15 | - | - | - | 15 | 4 | 15 | 15 | 10 | - | - | - | 1 | - |
|  |  |  |  |  |  |  |  |  |  |  |  |  |  |  |
| ***Europe*** |  |  |  |  |  |  |  |  |  |  |  |  |  |  |
| Germany | 12 | - | 1 | 1 | 10 | 3 | 1 | 1 | 1 | 1 | 1 | 1 | - | - |
| Kazakhstan | 1 | - | - | - | 1 | - | - | 1 | - | - | - | - | - | - |
| Portugal | 1 | - | - | - | 1 | 1 | 1 | 1 | - | - | - | - | - | - |
| Spain | 2 | - | - | - | 2 | 1 | - | 1 | - | - | - | - | - | - |
|  |  |  |  |  |  |  |  |  |  |  |  |  |  |  |
| ***South America*** | |  |  |  |  |  |  |  |  |  |  |  |  |  |
| Brazil | 7 | - | - | - | 7 | 2 | 4 | 4 | 2 | 1 | - | - | 1 | 1 |
| Colombia | 1 | - | - | - | 1 | - | 1 | 1 | - | - | - | - | - | - |
| Peru | 31 | - | 5 | - | 26 | 9 | 9 | 11 | 6 | 1 | - | 1 | 1 | - |
| Rep. Domin. | 1 | - | - | - | 1 | - | - | - | - | - | - | - | - | - |
|  |  |  |  |  |  |  |  |  |  |  |  |  |  |  |
| **Overall** | **144** | **10** | **33** | **4** | **97** | **48** | **73** | **61** | **41** | **8** | **4** | **6** | **9** | **6** |

CAR Central African Republic; DRC Democratic Republic of Congo, L1-L4 lineages 1 to 4, (first line drugs) RMP = Rifampicin, INH = Isoniazid, SM = Streptomycin, EMB = Ethambutol; (second line drugs) OFL = Ofloxacin , KAN = kanamycin, CAP = capreomycin, Et = ethionamide, P =Para-aminosalisylic acid.

**Table S2**

The isolate ENA accession numbers and MIC values

| ENA Accession | TDR Accession | RMP MIC | INH MIC | SM MIC | EMB MIC |
| --- | --- | --- | --- | --- | --- |
| ERR1213824 | TB-TDR-0070 | >120 | 3.2 | 4 | 8 |
| ERR1213825 | TB-TDR-0073 | >120 | 0.8 | ≤1 | 4 |
| ERR1213826 | TB-TDR-0074 | 80 | 3.2 | 2 | ≤1 |
| ERR1213827 | TB-TDR-0077 | 30 | 0.2 | 2 | ≤1 |
| ERR1213828 | TB-TDR-0078 | ≤10 | 0.2 | ≤1 | 2 |
| ERR1213829 | TB-TDR-0079 | 80 | 0.2 | 4 | ≤1 |
| ERR1213830 | TB-TDR-0080 | ≤10 | 0.2 | ≤1 | ≤1 |
| ERR1213831 | TB-TDR-0081 | ≤10 | 0.2 | ≤1 | 2 |
| ERR1213832 | TB-TDR-0082 | ≤10 | 3.2 | ≤1 | 2 |
| ERR1213833 | TB-TDR-0083 | ≤10 | 3.2 | ≤1 | ≤1 |
| ERR1213834 | TB-TDR-0084 | ≤10 | 3.2 | ≤1 | ≤1 |
| ERR1213835 | TB-TDR-0085 | ≤10 | 3.2 | 8 | 4 |
| ERR1213836 | TB-TDR-0086 | >120 | 0.8 | ≤1 | 2 |
| ERR1213837 | TB-TDR-0087 | >120 | 3.2 | 8 | 4 |
| ERR1213838 | TB-TDR-0088 | >120 | 0.2 | ≤1 | ≤1 |
| ERR1213839 | TB-TDR-0089 | >120 | 0.8 | ≤1 | 8 |
| ERR1213840 | TB-TDR-0090 | >120 | 0.2 | ≤1 | 2 |
| ERR1213841 | TB-TDR-0091 | 20 | 0.2 | 2 | 2 |
| ERR1213842 | TB-TDR-0092 | ≤10 | 0.2 | 2 | 4 |
| ERR1213843 | TB-TDR-0093 | ≤10 | >3.2 | 8 | 4 |
| ERR1213844 | TB-TDR-0094 | ≤10 | 0.2 | ≤1 | 8 |
| ERR1213845 | TB-TDR-0095 | ≤10 | 0.8 | >16 | ≤1 |
| ERR1213846 | TB-TDR-0096 | ≤10 | ≤0.05 | >16 | ≤1 |
| ERR1213847 | TB-TDR-0097 | 30 | 0.2 | >16 | ≤1 |
| ERR1213848 | TB-TDR-0098 | 40 | 0.2 | 16 | 2 |
| ERR1213849 | TB-TDR-0099 | >120 | >3.2 | 4 | >8 |
| ERR1213850 | TB-TDR-0101 | 80 | 3.2 | 2 | 4 |
| ERR1213851 | TB-TDR-0102 | >120 | 3.2 | ≤1 | 2 |
| ERR1213852 | TB-TDR-0104 | ≤10 | >3.2 | ≤1 | 4 |
| ERR1213853 | TB-TDR-0106 | >120 | 3.2 | ≤1 | 2 |
| ERR1213854 | TB-TDR-0108 | ≤10 | 0.8 | 8 | ≤1 |
| ERR1213855 | TB-TDR-0109 | ≤10 | 3.2 | >16 | 2 |
| ERR1213856 | TB-TDR-0110 | ≤10 | >3.2 | >16 | 2 |
| ERR1213857 | TB-TDR-0112 | >120 | 0.8 | >16 | ≤1 |
| ERR1213858 | TB-TDR-0113 | 120 | >3.2 | >16 | 4 |
| ERR1213859 | TB-TDR-0116 | 80 | >3.2 | 8 | >8 |
| ERR1213860 | TB-TDR-0117 | >120 | >3.2 | ≤1 | 2 |
| ERR1213861 | TB-TDR-0119 | >120 | 3.2 | 4 | ≤1 |
| ERR1213862 | TB-TDR-0120 | 30 | >3.2 | 16 | 4 |
| ERR1213863 | TB-TDR-0122 | >120 | 3.2 | >16 | 4 |
| ERR1213864 | TB-TDR-0123 | 20 | >3.2 | >16 | 8 |
| ERR1213865 | TB-TDR-0124 | >120 | >3.2 | >16 | 4 |
| ERR1213866 | TB-TDR-0125 | >120 | 0.2 | ≤1 | 2 |
| ERR1213867 | TB-TDR-0126 | ≤10 | 0.2 | 2 | ≤1 |
| ERR1213868 | TB-TDR-0129 | >120 | 1.6 | 2 | ≤1 |
| ERR1213869 | TB-TDR-0130 | ≤10 | ≤0.05 | 2 | ≤1 |
| ERR1213870 | TB-TDR-0131 | >120 | >3.2 | >16 | ≤1 |
| ERR1213871 | TB-TDR-0132 | ≤10 | >3.2 | 4 | 4 |
| ERR1213872 | TB-TDR-0133 | >120 | 1.6 | 8 | 4 |
| ERR1213873 | TB-TDR-0134 | >120 | >3.2 | 4 | ≤1 |
| ERR1213874 | TB-TDR-0135 | >120 | >3.2 | ≤1 | >8 |
| ERR1213875 | TB-TDR-0136 | ≤10 | ≤0.05 | 2 | ≤1 |
| ERR1213876 | TB-TDR-0137 | ≤10 | ≤0.05 | 2 | ≤1 |
| ERR1213877 | TB-TDR-0138 | ≤10 | ≤0.05 | 2 | 2 |
| ERR1213878 | TB-TDR-0139 | ≤10 | 0.2 | 2 | 2 |
| ERR1213879 | TB-TDR-0140 | ≤10 | 0.2 | ≤1 | ≤1 |
| ERR1213880 | TB-TDR-0141 | >120 | 0.2 | >16 | ≤1 |
| ERR1213881 | TB-TDR-0142 | 20 | 0.2 | 2 | 2 |
| ERR1213882 | TB-TDR-0143 | ≤10 | 0.2 | 16 | 2 |
| ERR1213883 | TB-TDR-0144 | ≤10 | >3.2 | >16 | 4 |
| ERR1213884 | TB-TDR-0146 | 40 | 0.2 | 8 | ≤1 |
| ERR1213885 | TB-TDR-0147 | 20 | >3.2 | >16 | ≤1 |
| ERR1213886 | TB-TDR-0148 | >120 | >3.2 | >16 | 2 |
| ERR1213887 | TB-TDR-0149 | >120 | >3.2 | >16 | 4 |
| ERR1213888 | TB-TDR-0150 | >120 | 1.6 | 2 | ≤1 |
| ERR1213889 | TB-TDR-0152 | 120 | 3.2 | 8 | 8 |
| ERR1213890 | TB-TDR-0153 | 80 | 3.2 | 4 | 2 |
| ERR1213891 | TB-TDR-0155 | 120 | >3.2 | 2 | 2 |
| ERR1213892 | TB-TDR-0156 | ≤10 | 0.2 | 2 | 4 |
| ERR1213893 | TB-TDR-0157 | >120 | 0.2 | >16 | 2 |
| ERR1213894 | TB-TDR-0158 | ≤10 | ≤0.05 | 2 | ≤1 |
| ERR1213895 | TB-TDR-0159 | ≤10 | ≤0.05 | 2 | ≤1 |
| ERR1213896 | TB-TDR-0160 | 80 | 0.2 | 8 | ≤1 |
| ERR1213897 | TB-TDR-0161 | ≤10 | ≤0.05 | ≤1 | ≤1 |
| ERR1213898 | TB-TDR-0163 | ≤10 | ≤0.05 | ≤1 | ≤1 |
| ERR1213899 | TB-TDR-0164 | ≤10 | ≤0.05 | 8 | ≤1 |
| ERR1213900 | TB-TDR-0165 | >120 | 0.2 | 2 | ≤1 |
| ERR1213901 | TB-TDR-0166 | >120 | 3.2 | >16 | 2 |
| ERR1213902 | TB-TDR-0167 | >120 | 3.2 | 16 | 4 |
| ERR1213903 | TB-TDR-0169 | ≤10 | ≤0.05 | >16 | ≤1 |
| ERR1213904 | TB-TDR-0170 | >120 | >3.2 | 2 | 4 |
| ERR1213905 | TB-TDR-0171 | >120 | 0.2 | ≤1 | ≤1 |
| ERR1213906 | TB-TDR-0172 | 20 | >3.2 | 2 | ≤1 |
| ERR1213907 | TB-TDR-0173 | ≤10 | ≤0.05 | ≤1 | ≤1 |
| ERR1213908 | TB-TDR-0174 | ≤10 | ≤0.05 | 2 | ≤1 |
| ERR1213909 | TB-TDR-0175 | >120 | 0.8 | ≤1 | 4 |
| ERR1213910 | TB-TDR-0176 | >120 | 1.6 | >16 | 2 |
| ERR1213911 | TB-TDR-0177 | 20 | 0.2 | 2 | ≤1 |
| ERR1213912 | TB-TDR-0178 | ≤10 | 0.8 | 2 | ≤1 |
| ERR1213913 | TB-TDR-0180 | ≤10 | 0.2 | 2 | ≤1 |
| ERR1213914 | TB-TDR-0181 | ≤10 | ≤0.05 | 8 | 2 |
| ERR1213915 | TB-TDR-0182 | 20 | ≤0.05 | >16 | ≤1 |
| ERR1213916 | TB-TDR-0183 | >120 | 0.8 | 2 | ≤1 |
| ERR1213917 | TB-TDR-0184 | ≤10 | ≤0.05 | 2 | ≤1 |
| ERR1213918 | TB-TDR-0185 | >120 | 0.8 | 2 | ≤1 |
| ERR1213919 | TB-TDR-0186 | >120 | >3.2 | ≤1 | ≤1 |
| ERR1213920 | TB-TDR-0187 | ≤10 | >3.2 | 8 | 2 |
| ERR1213921 | TB-TDR-0189 | >120 | >3.2 | 8 | ≤1 |
| ERR1213922 | TB-TDR-0190 | >120 | ≤0.05 | >16 | ≤1 |
| ERR1213923 | TB-TDR-0191 | >120 | >3.2 | 8 | 8 |
| ERR1213924 | TB-TDR-0193 | >120 | >3.2 | >16 | 2 |
| ERR1213925 | TB-TDR-0194 | 20 | 0.2 | 2 | ≤1 |
| ERR1213926 | TB-TDR-0195 | 20 | 3.2 | 2 | 8 |
| ERR1213927 | TB-TDR-0197 | 20 | 0.2 | ≤1 | 2 |
| ERR1213928 | TB-TDR-0198 | >120 | 3.2 | 8 | 4 |
| ERR1213929 | TB-TDR-0199 | ≤10 | 0.2 | ≤1 | 2 |
| ERR1213930 | TB-TDR-0200 | ≤10 | ≤0.05 | ≤1 | ≤1 |
| ERR1213931 | TB-TDR-0201 | >120 | >3.2 | 16 | ≤1 |
| ERR1213932 | TB-TDR-0202 | 20 | ≤0.05 | 4 | 2 |
| ERR1213933 | TB-TDR-0203 | ≤10 | ≤0.05 | 2 | ≤1 |
| ERR1213934 | TB-TDR-0204 | 20 | ≤0.05 | 4 | ≤1 |
| ERR1213935 | TB-TDR-0207 | ≤10 | ≤0.05 | 2 | ≤1 |
| ERR1213936 | TB-TDR-0208 | 20 | 0.2 | ≤1 | ≤1 |
| ERR1213937 | TB-TDR-0209 | 20 | 0.2 | 2 | ≤1 |
| ERR1213938 | TB-TDR-0210 | 20 | ≤0.05 | ≤1 | ≤1 |
| ERR1213939 | TB-TDR-0213 | 20 | 0.8 | 2 | ≤1 |
| ERR1213940 | TB-TDR-0214 | ≤10 | ≤0.05 | 2 | ≤1 |
| ERR1213941 | TB-TDR-0016 | ≤10 | 0.2 | ≤1 | 2 |
| ERR1213942 | TB-TDR-0017 | ≤10 | 0.2 | 2 | ≤1 |
| ERR1213943 | TB-TDR-0018 | >120 | 0.2 | ≤1 | ≤1 |
| ERR1213944 | TB-TDR-0022 | 20 | ≤0.05 | 2 | ≤1 |
| ERR1213945 | TB-TDR-0038 | ≤10 | >3.2 | >16 | ≤1 |
| ERR1213946 | TB-TDR-0041 | ≤10 | ≤0.05 | 2 | ≤1 |
| ERR1213947 | TB-TDR-0042 | ≤10 | >3.2 | ≤1 | >8 |
| ERR1213948 | TB-TDR-0043 | 20 | 3.2 | ≤1 | 8 |
| ERR1213949 | TB-TDR-0045 | ≤10 | 0.2 | 2 | 4 |
| ERR1213950 | TB-TDR-0007 | >120 | >3.2 | >16 | 4 |

RMP rifampicin, INH isoniazid, SM streptomycin, EMB ethambutol

**Table S3**

Drug susceptibility profiles for rifampicin, isoniazid, streptomycin and ethambutol

| No. samples | Rifampicin | Isoniazid | Streptomycin | Ethambutol |
| --- | --- | --- | --- | --- |
| *12 (9.4%)* | ***R*** | ***R*** | ***R*** | ***R*** |
| *8 (6.3%)* | ***R*** | ***R*** | ***R*** | *S* |
| *8 (6.3%)* | ***R*** | ***R*** | *S* | ***R*** |
| *14 (11.0%)* | ***R*** | ***R*** | *S* | *S* |
| 4 (3.1%) | **R** | S | **R** | S |
| 7 (5.5%) | **R** | S | S | S |
| 5 (3.4%) | S | **R** | **R** | **R** |
| 7 (5.5%) | S | **R** | **R** | S |
| 5 (3.4%) | S | **R** | S | **R** |
| 6 (4.7%) | S | **R** | S | S |
| 9 (7.1%) | S | S | **R** | **S** |
| 4 (3.1%) | S | S | S | **R** |
| 38 (29.9%) | S | S | S | S |

R = resistance, S = sensitive; 13 different profiles were identified across 127 independent samples; Multi-drug resistant in italics

**Table S4**

Combinations of mutations and their frequency (N) in drug resistance candidate genes

a) Rifampicin

| Mutation observed in *rpoB* codons | | | | | | | | | | *rpoC* | N | MIC (µg/ml) | | |
| --- | --- | --- | --- | --- | --- | --- | --- | --- | --- | --- | --- | --- | --- | --- |
| 45 | 170 | 250 | 400 | 435 | 445 | 4  5  0 | 491 | 692 | rare |  |  | Mean | Min | Max |
|  |  |  |  |  |  |  |  |  |  |  | 70 | 15.3 | 10.0 | 80.0 |
|  |  |  |  |  |  |  |  |  | * |  | 5 | 68.0 | 10.0 | 120.0 |
|  |  |  |  |  |  |  |  | * |  |  | 1 | 10.0 | 10.0 | 10.0 |
|  |  |  |  |  |  |  | * |  |  |  | 1 | 80.0 | 80.0 | 80.0 |
|  |  |  |  |  |  | * |  |  |  |  | 21 | 114.3 | 80.0 | 120.0 |
|  |  |  |  |  |  | * |  |  |  | * | 1 | 120.0 | 120.0 | 120.0 |
|  |  |  |  |  |  | * |  |  | * |  | 2 | 120.0 | 120.0 | 120.0 |
|  |  |  |  |  |  | * |  |  | * | * | 1 | 120.0 | 120.0 | 120.0 |
|  |  |  |  |  |  | * | * |  |  |  | 1 | 120.0 | 120.0 | 120.0 |
|  |  |  |  |  |  | ** |  |  |  |  | 1 | 120.0 | 120.0 | 120.0 |
|  |  |  |  |  | * |  |  |  |  |  | 9 | 120.0 | 120.0 | 120.0 |
|  |  |  |  | * |  |  |  |  |  |  | 4 | 120.0 | 120.0 | 120.0 |
|  |  |  |  | * |  |  |  | * |  |  | 1 | 120.0 | 120.0 | 120.0 |
|  |  |  | * |  |  | * |  |  |  |  | 2 | 120.0 | 120.0 | 120.0 |
|  |  | * |  |  |  |  |  |  |  |  | 2 | 10.0 | 10.0 | 10.0 |
|  | * |  |  |  |  |  |  |  |  |  | 1 | 120.0 | 120.0 | 120.0 |
|  | * |  |  |  |  |  |  |  | * |  | 1 | 120.0 | 120.0 | 120.0 |
| * |  |  |  |  |  | * |  |  |  |  | 3 | 120.0 | 120.0 | 120.0 |

b) Isoniazid

| *katG* codons | | | *inhA*  prom. | N | MIC (µg/ml) | | |
| --- | --- | --- | --- | --- | --- | --- | --- |
| 315 | 436 | rare |  |  | Mean | Min | Max |
|  |  |  |  | 46 | 0.3 | 0.05 | 3.2 |
|  |  | * |  | 2 | 0.2 | 0.2 | 0.2 |
|  |  | *** |  | 1 | 3.2 | 3.2 | 3.2 |
|  |  |  | * | 8 | 1.7 | 0.8 | 3.2 |
|  |  | * | * | 1 | 1.6 | 1.6 | 1.6 |
| * |  |  |  | 23 | 2.9 | 0.2 | 3.2 |
| * |  | * |  | 1 | 3.2 | 3.2 | 3.2 |
| * |  |  | * | 2 | 3.2 | 3.2 | 3.2 |
|  | * |  |  | 18 | 0.2 | 0.05 | 0.8 |
|  | * |  | * | 4 | 1.0 | 0.8 | 1.6 |
|  | * | * | * | 1 | 3.2 | 3.2 | 3.2 |
| * | * |  |  | 18 | 3.2 | 3.2 | 3.2 |
| * | * | * |  | 1 | 3.2 | 3.2 | 3.2 |
| * | * |  | * | 1 | 3.2 | 3.2 | 3.2 |

**c)** Streptomycin

| *rpsL* codons | | *rrs* | N | MIC (µg/ml) | | |
| --- | --- | --- | --- | --- | --- | --- |
| 43 | 88 |  |  | Mean | Min | Max |
|  |  |  | 99 | 3.8 | 1 | 16 |
|  |  | * | 13 | 10.0.4 | 1 | 16 |
|  | * |  | 4 | 16.0 | 16 | 16 |
| * |  |  | 11 | 16.0 | 16 | 16 |

**d)** Ethambutol

| *embB* codons | | | | | |  |  |  | *ubiA* | *embA* | N | MIC (µg/ml) | | |
| --- | --- | --- | --- | --- | --- | --- | --- | --- | --- | --- | --- | --- | --- | --- |
| 297 | 306 | 319 | 354 | 378 | 406 | 497 | 1024 | r  a  r  e |  |  |  | Mean | Min | Max |
|  |  |  |  |  |  |  |  |  |  |  | 70 | 1.2 | 1 | 2 |
|  |  |  |  |  |  |  |  |  |  | ** | 1 | 4.0 | 4 | 4 |
|  |  |  |  |  |  |  |  |  | * |  | 2 | 1.5 | 1 | 2 |
|  |  |  |  |  |  |  |  |  | ** |  | 1 | 1.0 | 1 | 1 |
|  |  |  |  |  |  |  |  | * |  |  | 3 | 1.7 | 1 | 2 |
|  |  |  |  |  |  |  |  | ** |  |  | 1 | 2.0 | 2 | 2 |
|  |  |  |  |  |  |  | * |  |  |  | 1 | 4.0 | 4 | 4 |
|  |  |  |  |  |  | * |  |  |  |  | 5 | 4.8 | 4 | 8 |
|  |  |  |  |  |  | * |  |  | * |  | 1 | 4.0 | 4 | 4 |
|  |  |  |  |  | * |  |  |  |  |  | 6 | 4.5 | 1 | 8 |
|  |  |  |  |  | * |  |  |  | * |  | 4 | 2.8 | 1 | 4 |
|  |  |  |  |  | * |  | * |  |  |  | 1 | 4.0 | 4 | 4 |
|  |  |  |  |  | * |  | * | * |  |  | 1 | 4.0 | 4 | 4 |
|  |  |  |  | * |  |  |  |  | ** |  | 3 | 1.0 | 1 | 1 |
|  |  |  |  | * |  | * |  | * | ** |  | 1 | 2.0 | 2 | 2 |
|  |  |  | * |  |  |  |  |  |  | * | 1 | 4.0 | 4 | 4 |
|  |  |  | * | * |  |  |  |  | ** |  | 1 | 4.0 | 4 | 4 |
|  |  |  | * | * |  |  |  | * | ** |  | 1 | 4.0 | 4 | 4 |
|  |  | * |  |  |  |  |  |  |  |  | 3 | 4.7 | 2 | 8 |
|  | * |  |  |  |  |  |  |  |  |  | 11 | 4.1 | 1 | 8 |
|  | * |  |  |  |  |  |  |  |  | * | 1 | 4.0 | 4 | 4 |
|  | * |  |  |  |  |  |  |  | * |  | 1 | 8.0 | 8 | 8 |
|  | * |  |  |  |  |  |  | * |  |  | 1 | 2.0 | 2 | 2 |
|  | * |  |  | * |  |  |  |  | ** |  | 2 | 8.0 | 8 | 8 |
|  | * |  | * |  |  |  |  |  |  |  | 1 | 8.0 | 8 | 8 |
| * |  |  |  |  |  |  |  |  |  |  | 2 | 2.5 | 1 | 4 |
| * |  |  |  |  |  |  |  |  |  | * | 1 | 8.0 | 8 | 8 |

* single mulation, ** double mutations, *** triple mutations; SNP mutations in a single sample have been aggregated into a “rare” column.

**Table S5**

Predicted effects of mutations

| Gene | Mutation | Distance to interface (Å) | Distance to ligand (Å) | DUET (ΔΔG kcal/mol) | mCSM-Stability (ΔΔG kcal/mol) | SDM (ΔΔG kcal/mol) |
| --- | --- | --- | --- | --- | --- | --- |
| *rpoB* | T400A |  | 42.914 | 0.031 | -0.326 | 2.480 |
|  | D435V |  | 3.094 | 0.336 | 0.356 | 1.860 |
|  | H445D |  | 4.015 | -2.084 | -1.971 | -1.730 |
|  | H445Y |  | 4.015 | -0.214 | -0.171 | -0.310 |
|  | H445R |  | 4.015 | -1.958 | -1.857 | -1.950 |
|  | S450W |  | 5.773 | -0.756 | -0.840 | 2.330 |
|  | S450L |  | 5.773 | 0.102 | -0.126 | 2.820 |
|  | I491V |  | 2.908 | -1.221 | -1.274 | -0.830 |
|  | I491F |  | 2.908 | -1.529 | -1.416 | -0.760 |
| *katG* | S315N | 14.940 | 2.149 | -0.100 | -0.184 | 2.149 |
|  | S315T | 14.940 | 2.149 | -0.243 | -0.330 | 2.149 |
