# Supplementary figures and images for: Mycobacterium tuberculosis whole genome sequencing and protein structure modelling provides insights into anti-tuberculosis drug resistance
Source: BMC Med. 2016 Mar 23;14:31. doi: 10.1186/s12916-016-0575-9 (PMC4804620; doi:10.1186/s12916-016-0575-9)

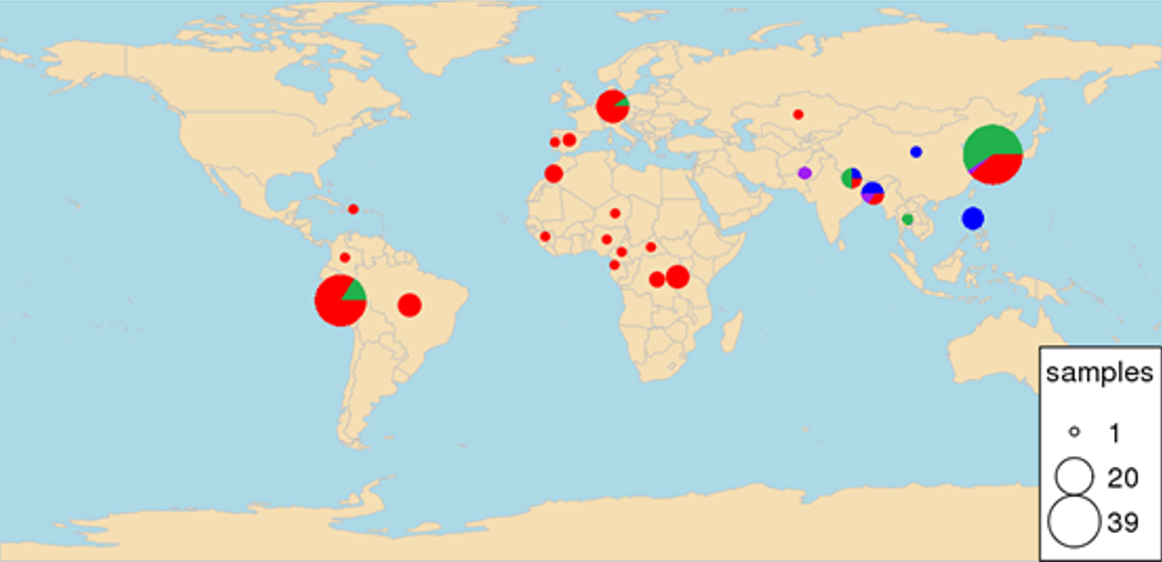

Supplement: Additional file 2: Figure S1. — The global distribution of geographic origin and lineage of the isolates. Lineages one to four are represented by blue, green, purple, and red, respectively. (PNG 265 kb) [file 12916_2016_575_MOESM2_ESM.png]

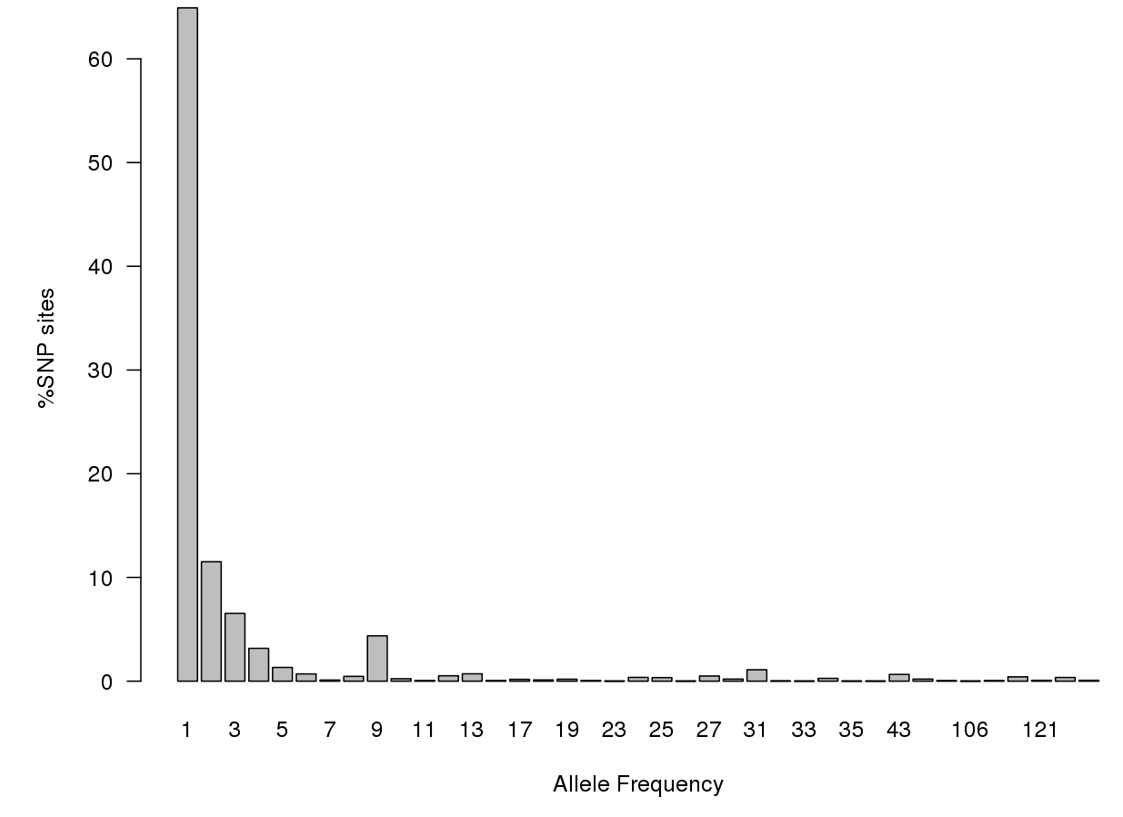

Supplement: Additional file 3: Figure S2. — SNP allele frequency spectrum. A large number of rare variants are observed. Peaks with higher allele frequency reflect the presence of lineage and sub-lineage specific SNPs. (PNG 33 kb) [file 12916_2016_575_MOESM3_ESM.png]

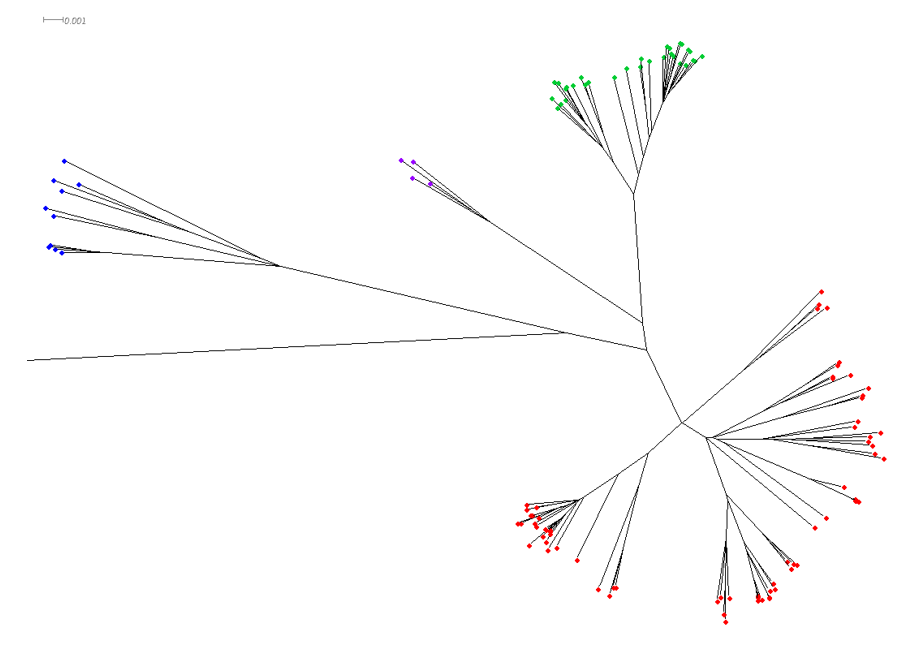

Supplement: Additional file 4: Figure S3. — Population structure analysis of the 144 isolates show clustering by lineage (Lineages one to four are represented by blue, green, purple, and red points, respectively). (a) A phylogenetic tree rooted with M. canetti. (b) First two principal components represent 33 % and 30.5 % of the variation explained between isolates, respectively. (ZIP 105 kb) [file 12916_2016_575_MOESM4_ESM.zip › SuppFig3/SuppFig3aR2.png]

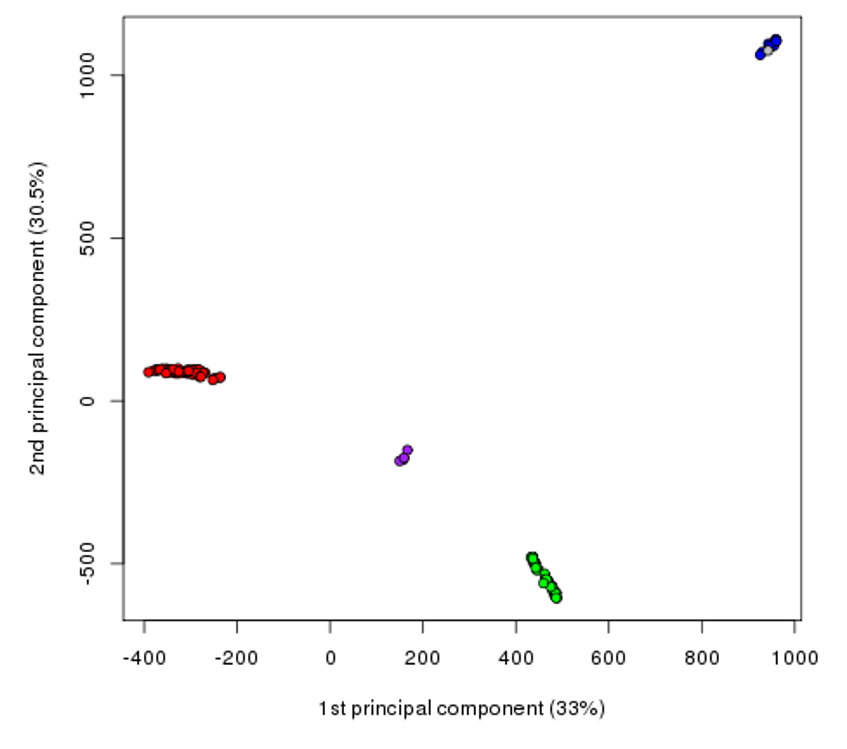

Supplement: Additional file 4: Figure S3. — Population structure analysis of the 144 isolates show clustering by lineage (Lineages one to four are represented by blue, green, purple, and red points, respectively). (a) A phylogenetic tree rooted with M. canetti. (b) First two principal components represent 33 % and 30.5 % of the variation explained between isolates, respectively. (ZIP 105 kb) [file 12916_2016_575_MOESM4_ESM.zip › SuppFig3/SuppFig3bR2.png]

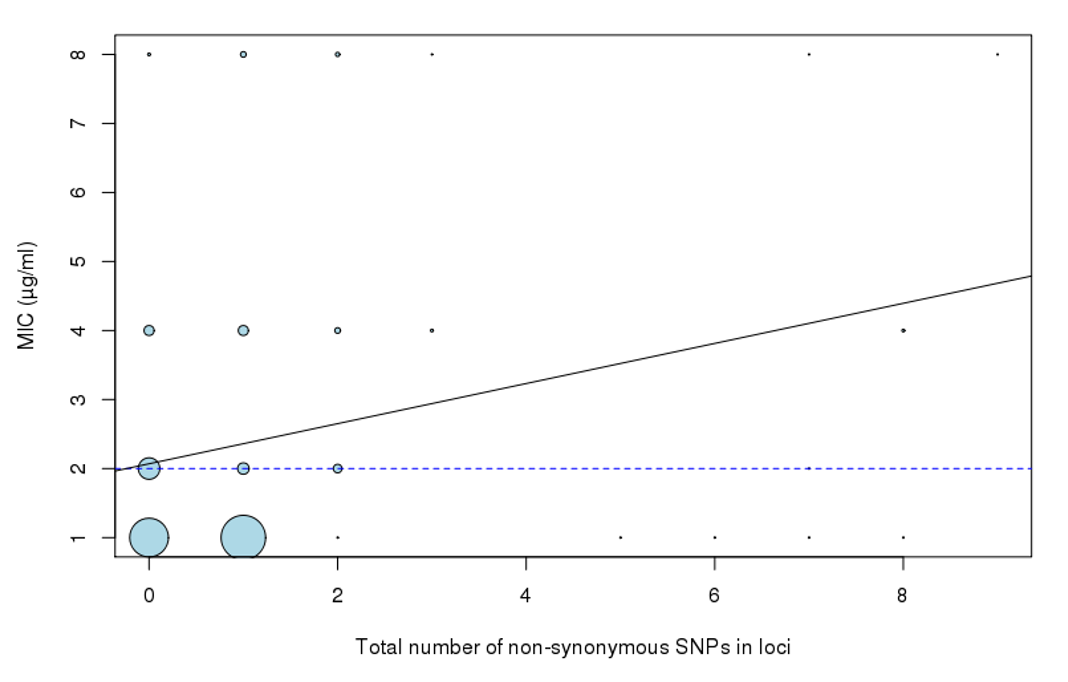

Supplement: Additional file 5: Figure S4. — The relationship between the total number of non-synonymous SNPs in candidate loci and the MIC values. The size of the circle represents the number of isolates. a) Ethambutol (embB, embA, embA promoter, embC, embR and ubiA). b) Streptomycin (rpsL, rrs). The size of the circles is proportional to the frequency. The MIC values tend to increase with the number of non-synonymous mutations (ethambutol: rho = 0.24, slope = 0.29, p = 0.003; streptomycin: rho = 0.48, slope = 3.59, p = 1.65 × 10^-8^). The horizontal blue lines refer to the resistance cut-offs. (ZIP 92 kb) [file 12916_2016_575_MOESM5_ESM.zip › SuppFig4/SuppFig4aR2.png]

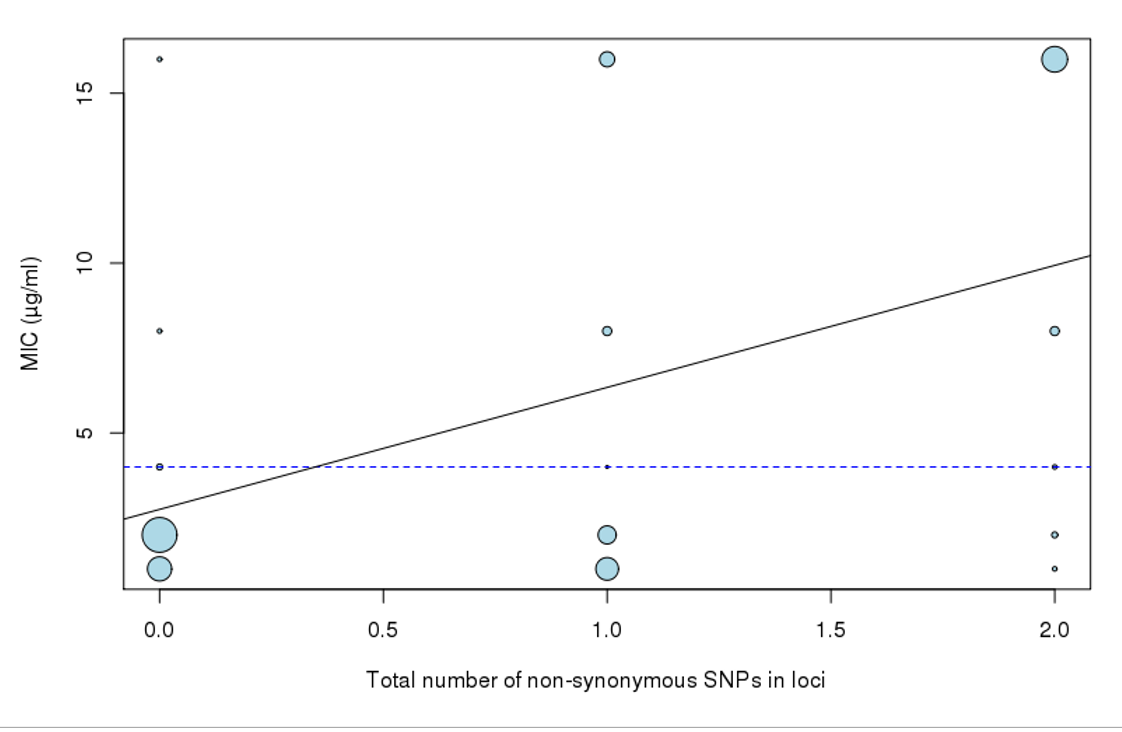

Supplement: Additional file 5: Figure S4. — The relationship between the total number of non-synonymous SNPs in candidate loci and the MIC values. The size of the circle represents the number of isolates. a) Ethambutol (embB, embA, embA promoter, embC, embR and ubiA). b) Streptomycin (rpsL, rrs). The size of the circles is proportional to the frequency. The MIC values tend to increase with the number of non-synonymous mutations (ethambutol: rho = 0.24, slope = 0.29, p = 0.003; streptomycin: rho = 0.48, slope = 3.59, p = 1.65 × 10^-8^). The horizontal blue lines refer to the resistance cut-offs. (ZIP 92 kb) [file 12916_2016_575_MOESM5_ESM.zip › SuppFig4/SuppFig4bR2.png]

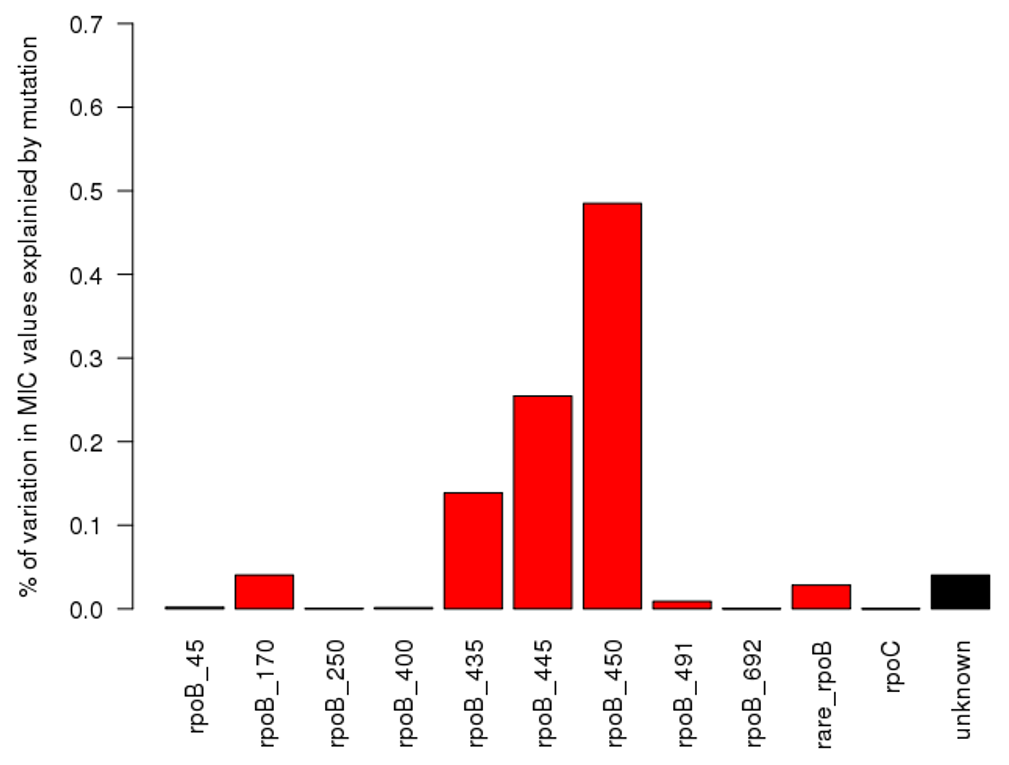

Supplement: Additional file 6: Figure S5. — Percentage of the variation in MIC values explained by each mutated codon in candidate genes. Bars in red represent significant independent associations with increased MIC (p < 0.05). a) Rifampicin. b) Isoniazid. c) Streptomycin. d) Ethambutol. (ZIP 231 kb) [file 12916_2016_575_MOESM6_ESM.zip › SuppFig5/SuppFig5aR2.png]

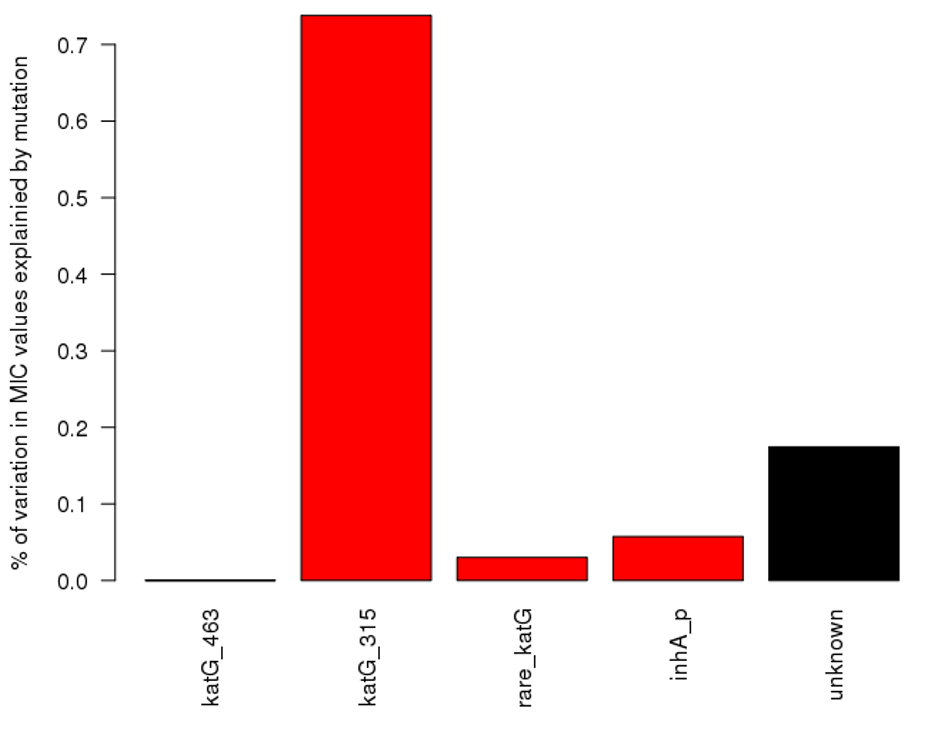

Supplement: Additional file 6: Figure S5. — Percentage of the variation in MIC values explained by each mutated codon in candidate genes. Bars in red represent significant independent associations with increased MIC (p < 0.05). a) Rifampicin. b) Isoniazid. c) Streptomycin. d) Ethambutol. (ZIP 231 kb) [file 12916_2016_575_MOESM6_ESM.zip › SuppFig5/SuppFig5bR2.png]

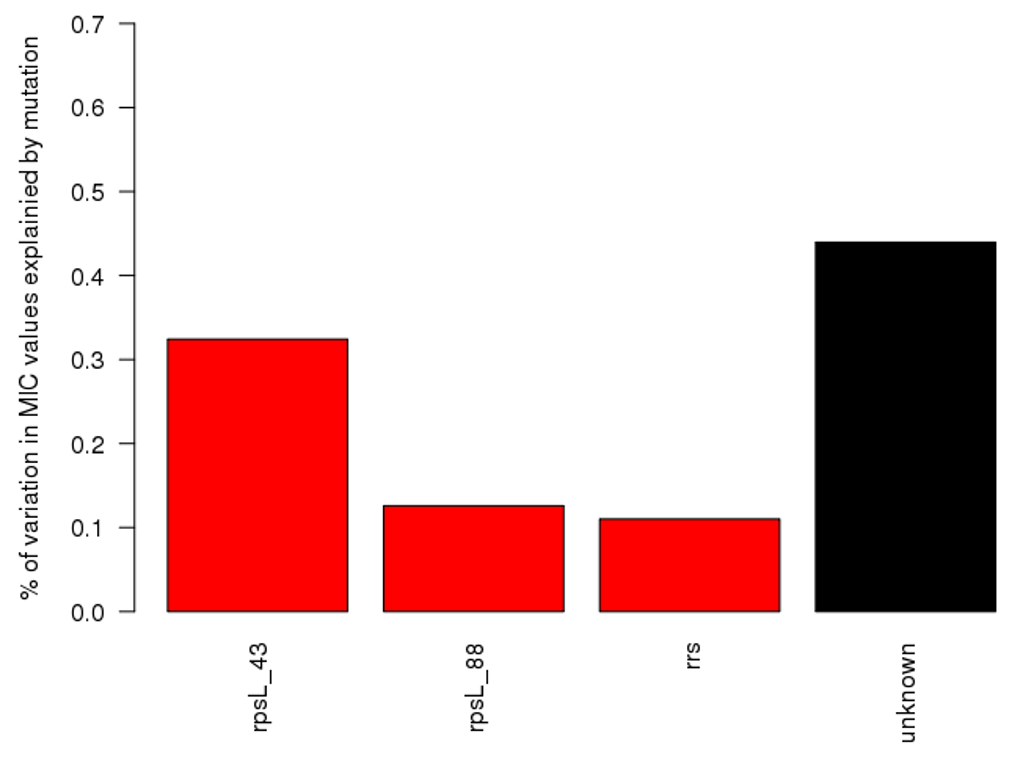

Supplement: Additional file 6: Figure S5. — Percentage of the variation in MIC values explained by each mutated codon in candidate genes. Bars in red represent significant independent associations with increased MIC (p < 0.05). a) Rifampicin. b) Isoniazid. c) Streptomycin. d) Ethambutol. (ZIP 231 kb) [file 12916_2016_575_MOESM6_ESM.zip › SuppFig5/SuppFig5cR2.png]

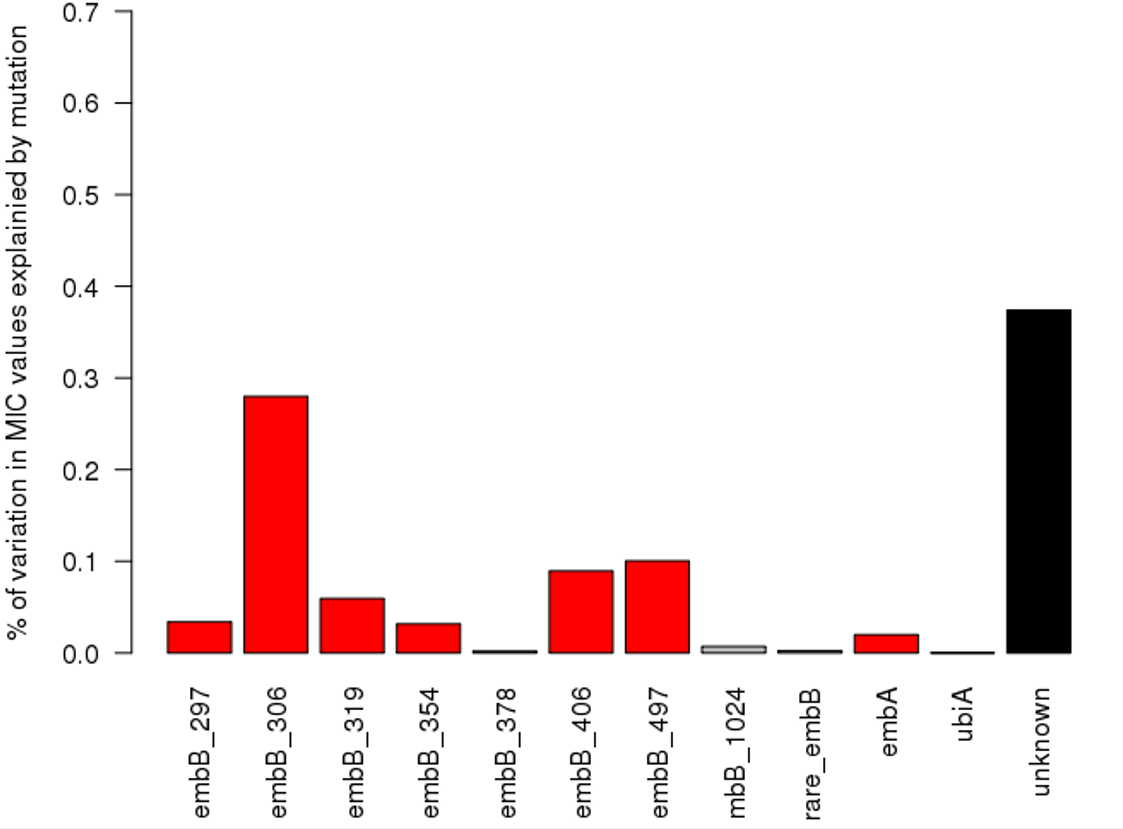

Supplement: Additional file 6: Figure S5. — Percentage of the variation in MIC values explained by each mutated codon in candidate genes. Bars in red represent significant independent associations with increased MIC (p < 0.05). a) Rifampicin. b) Isoniazid. c) Streptomycin. d) Ethambutol. (ZIP 231 kb) [file 12916_2016_575_MOESM6_ESM.zip › SuppFig5/SuppFig5dR2.png]

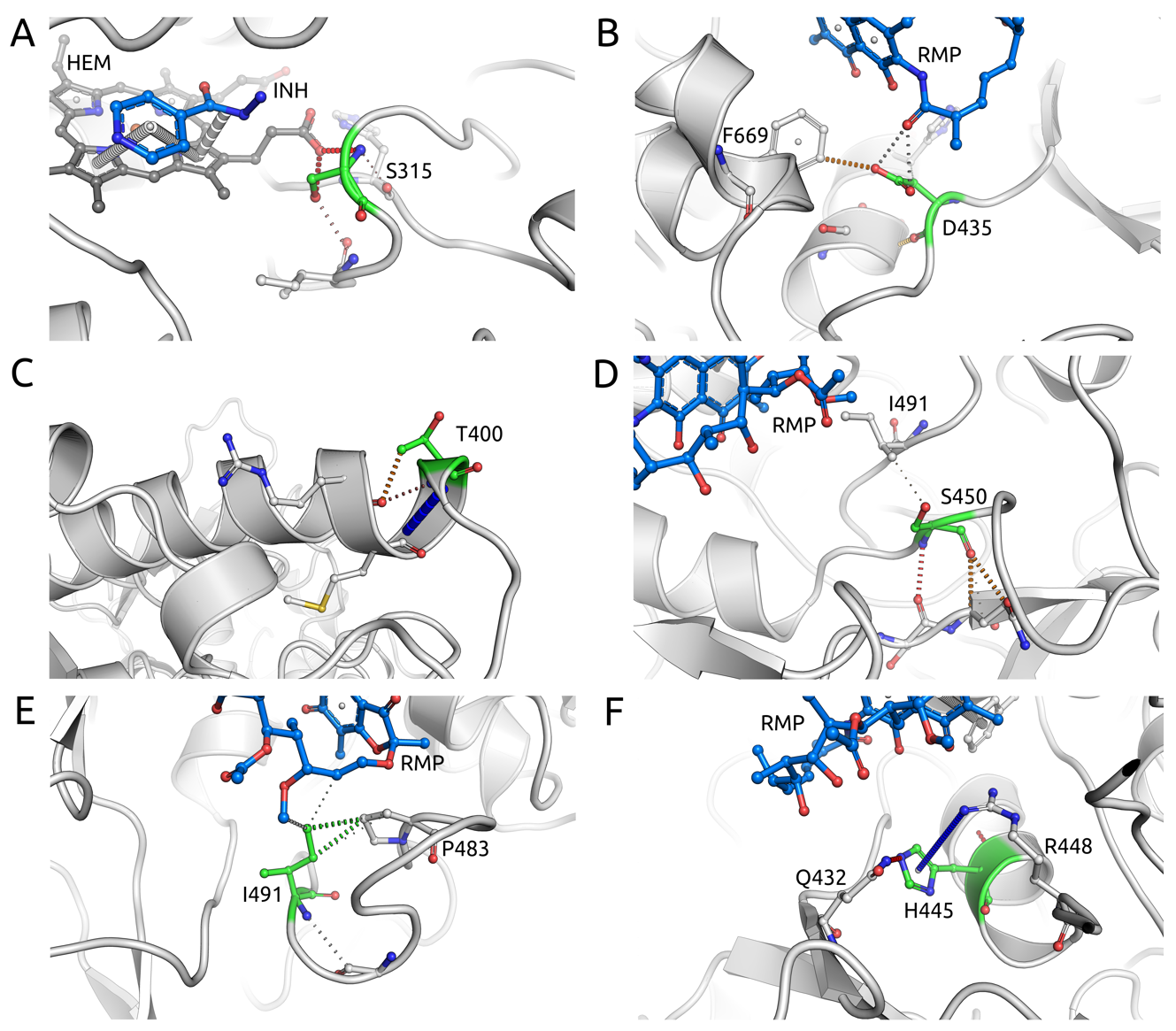

Supplement: Additional file 7: Figure S6. — Molecular interactions established by wild-type residues in katG and rpoB residues. (A) The interactions established by Ser315 in katG. Given the proximity of the residue to the ligands INH and HEM, mutations to Asn and Thr, with slightly larger side chains, would potentially cause steric clashes. (B) The interactions of Asp435 in rpoB. It directly interacts with RMP via polar interactions that would be disrupted by mutations to Val. (C) Thr400 in rpoB is at the end of an alpha helix establishing intra molecular interactions. Giving its distance to RMP, it would be expected that its mutation to Ala would be a lower impact, which would arise from alosteric changes. (D) Ser450 establishes strong intra molecular interactions in the RMP binding site. Mutations to larger residues (Trp and Leu) could disrupt the packing of the region and therefore binding. (E). Ile491 performs hydrophobic interactions with RMP and its neighbouring residues. Mutations to Phe or Val would compromise packing, either inducing steric clashes or compromising packing. (F). His445 performs strong intra molecular interactions, including a donor-pi (blue dashes) and hydrogen bond (red dashes). Mutations to residues Asp, Tyr or Arg would imply in the loss of the pi interaction as well as potential introduction of steric clashes. (PNG 749 kb) [file 12916_2016_575_MOESM7_ESM.png]
